# Sweet Modifications Modulate Plant Development

**DOI:** 10.3390/biom11050756

**Published:** 2021-05-18

**Authors:** Tibo De Coninck, Koen Gistelinck, Henry C. Janse van Rensburg, Wim Van den Ende, Els J. M. Van Damme

**Affiliations:** 1 Laboratory of Glycobiology & Biochemistry, Department of Biotechnology, Ghent University, Coupure Links 653, B-9000 Ghent, Belgium; tibo.deconinck@ugent.be (T.D.C.); koen.gistelinck@ugent.be (K.G.); 2 Laboratory of Molecular Plant Biology, Department of Biology, KU Leuven, Kasteelpark Arenberg 31, B-3001 Leuven, Belgium; henry.jansevanrensburg@unibas.ch (H.C.J.v.R.); wim.vandenende@kuleuven.be (W.V.d.E.)

**Keywords:** glycosylation, glycoproteins, glycolipids, plant development, signaling, sugars

## Abstract

Plant development represents a continuous process in which the plant undergoes morphological, (epi)genetic and metabolic changes. Starting from pollination, seed maturation and germination, the plant continues to grow and develops specialized organs to survive, thrive and generate offspring. The development of plants and the interplay with its environment are highly linked to glycosylation of proteins and lipids as well as metabolism and signaling of sugars. Although the involvement of these protein modifications and sugars is well-studied, there is still a long road ahead to profoundly comprehend their nature, significance, importance for plant development and the interplay with stress responses. This review, approached from the plants’ perspective, aims to focus on some key findings highlighting the importance of glycosylation and sugar signaling for plant development.

## 1. Plant Development and Sugars

### 1.1. Plant Development in a Nut Shell

Plant development is an overarching term for a plethora of processes, including embryonal development, seed maturation and germination, and growth of the vegetative plant with specialized roots, shoots, leaves and flowers [1,2,3]. It has been shown that sugars, glycoproteins and glycolipids play a crucial role in various pathways such as hormone signaling, cellular trafficking, development and growth [4,5,6,7,8].

Seed development is universal and includes embryogenesis, maturation and germination. Embryogenesis follows after flower pollination and ovule fertilization by pollen grains, and is considered as the beginning of every plant’s life cycle [9]. The process starts with sequential coordinated cell divisions of the zygote, leading to a globular embryo, a cluster of undifferentiated cells. During embryogenesis, the evolving embryo receives a continuous flow of nutrients from the parental plant [10]. Later on, the primary meristems develop. Cell divisions in the protoderm and in the embryo lead to the development of the future epidermis on the one hand, and the ground meristem and procambium on the other hand, which in turn will develop to ground tissue and vascular tissue, respectively [9,11].

When the cotyledons have been established, the embryo is completely mature and the seed enters the maturation stage [12]. Seed coat hardening, together with the dehydration of the seed, characterizes the maturation stage and ensures viability during the subsequent dormancy phase [13]. Transition to the dormancy phase is heavily influenced by the levels of abscisic acid (ABA) vs. gibberellic acid (GA), produced by the developing zygote [14]. Before entering dormancy, the seed is dispersed as a consequence of fruit dehiscence and seed abscission [15,16]. Dormancy comes to an end when seed-dependent and environmental parameters are aligned. Just like entering, stepping out of the dormant phase, also called germination, is heavily regulated by the ABA/GA balance [17]. In this perspective, the uptake of water is of importance as it hydrates the aleurone layer and starchy endosperm. The hydrated aleurone layer releases GA, triggering the ab initio synthesis of enzymes, such as glycoside hydrolases (GHs) and proteases, breaking down the hydrated endosperm and loosening the cell wall. This causes a detrimental loss of internal integrity as well as the release of storage molecules, which are transferred to the growing seedling [18,19].

As germination proceeds, the primary root protrudes the enzymatically weakened seed coat, lateral roots and the hypocotyl emerge, and the cotyledons’ storage food gets depleted [20]. The seedling continues to develop through cellular growth, morphogenesis and differentiation. Growth is achieved by cell divisions and cellular elongation [21,22,23]. Both sugars and glycoproteins are of crucial importance during these steps [4,7,24]. As cell divisions pursue and the plant assumes a certain shape and form, environmental parameters cause the plant to further differentiate and develop. Apical shoot and root meristematic activity give rise to specialized organs such as roots, shoots, leaves and flowers. The seedling is now an autonomous photosynthesizing organism, in an environment prone to biotic and abiotic stresses.

Glycoproteins, glycolipids and sugars are heavily involved in mediating environmental cues [4,6,25,26,27]. During the course of a plant’s life cycle, cells undergo a multitude of changes: plant cells develop, grow and gain cellular volume [28]. As soon as germination is initiated, the cells of the growing hypocotyl, radicle and cotyledons multiply fast. This rapid growth requires intense enzymatic as well as hormonal control mechanisms [22].

During plant development, additional reorganization of cell wall constituents occurs to ensure optimal growth, adhesion, cellular expansion, but also defense against pathogens. The process of cellular expansion occurs through unidirectional tip growth (i.e., in root hairs, pollen tubes, trichomes and fibers) or polydirectional anisotropic growth (i.e., in most cells and tissues) [29]. The currently accepted mechanism of cell elongation states that the orientation of cell wall constituents determines the direction of expansion [29,30]. Cellular expansion relies on disassembly of the cell wall constituents, on one hand, and the outward directing force originating from turgor pressure on the other hand. This active process is driven by the disassembly of cell wall constituents, which causes the cell wall to loosen and relax, thereby allowing turgor pressure to affect the orientation of the loosened cell wall polysaccharides. Next, cellular expansion and growth, and the rupture of cell–cell interactions (i.e., abscission) of flowers, fruits, seeds and leaves are accompanied by structural changes and cell wall modifications [31,32,33,34].

To accommodate these physiological processes, plant cell walls need to be dynamic. In fact, there is a continuous equilibrium of assembly and disassembly of the cell wall constituents [34]. This balance is controlled by hormonal and environmental cues [31,35,36,37]. At status quo, the assembly–disassembly equilibrium is in balance, while cell wall dynamics can be skewed towards assembly or disassembly when enduring drastic environmental changes or when exposed to pathogenic attack. Alterations of the cell wall are mainly accommodated through carbohydrate-active enzymes (CAZymes; www.cazy.org (accessed on 1 April 2021)) as well as structural glycoproteins such as extensins (EXTs) and expansins [38].

### 1.2. Carbohydrate Classes: Free Saccharides and Glycoconjugates

Carbohydrates are biomolecules mainly consisting of C, H and O atoms. They can be divided into the free saccharides and the glycoconjugates. The free saccharides, commonly referred to as ‘sugars’, occur in many forms such as monosaccharides (e.g., hexoses), disaccharides (e.g., sucrose (Suc), maltose and trehalose), oligosaccharides (degree of polymerization: DP 3–9) and polysaccharides (DP ≥ 10), also termed glycans (e.g., glucans, fructans), which are covalently linked through glycosidic bonds [39]. Some of these polysaccharides remain water soluble (e.g., fructans) and are readily available for rapid enzymatic remobilization of their monosaccharidic substituents, while others are condensed in water insoluble entities (e.g., starch, glucans, in granules) requiring more time to release their (energetic) monosaccharides [5,40]. Besides acting as energy storage reserves and building blocks, small soluble saccharides like glucose (Glc), fructose (Fru) and Suc can also act as signaling entities modulating plant growth and stress responses [41]. Referring to the concept of Sweet Immunity, stress-mediated imbalance in plant source–sink relationships causes temporal mature leaf sweetening, contributing to the synthesis of antimicrobial compounds, some of them perhaps also able to counteract abiotic stresses [42]. Evidence is accumulating that extracellular spraying of carbohydrates (e.g., priming with fructans) changes the intracellular sugar signaling context, leading to disease protection [43].

**Figure 1:**
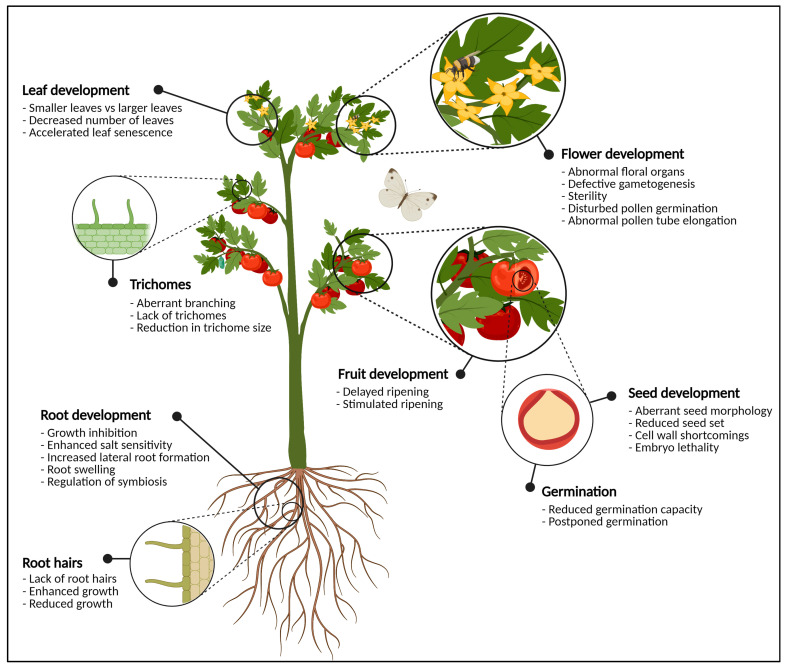
Graphical representation of the most important plant organs. The plant in this figure is generic and does not necessarily represent tomato. For each organ, the most significant phenotypical traits when glycosylation enzymes are knocked-out, knocked-down or over-expressed are highlighted. In the flowering parts, defects in glycosylation cause abnormal development of anthers and pistils, pollen tube germination and elongation, and defective gametogenesis which often leads to sterility. At the level of the fruit, glycosylation enzymes are important for fruit ripening and softening. Seeds show aberrant seed morphology, seed set, (embryo) lethality and cell wall defects when the expression of one or more glycosylation enzymes is disturbed. Vegetative tissues such as the leaves and the roots also experience morphological changes due to aberrant protein glycosylation. This figure was created with BioRender.com.

The glycoconjugates are composed of glycan structures which are covalently linked either co- or post-translationally to other non-sugar biomolecules such as peptides, proteins or lipids [44]. In general, the addition of a carbohydrate moiety to a protein or lipid is referred to as glycosylation. The process of protein glycosylation is considered the most complicated but ubiquitous modification of secretory proteins [45], the main types of glycosylation being N-, O-, P-, S- and C-glycosylation, referring to the atom which is involved in the glycosidic linkage [46]. However, N- and O-glycosylation are the most abundant in plants. It is estimated that approximately 50% of all proteins are glycoproteins, of which the majority is N-glycosylated [47,48]. The presence or absence of N- and O-glycans on glycoproteins has been shown to influence a proteins’ activity, stability and functionality to a large extent, and plays a critical role in cellular signaling, molecular trafficking, plant development and adaptation to biotic and abiotic stresses [7,49,50] (Figure 1). The ubiquity and importance of protein glycosylation is demonstrated by its wide evolutionary distribution across all kingdoms of life [51,52]. N-glycosylation is highly conserved, while O-glycosylation is less straightforward and greatly differs between various organisms of different kingdoms. The processes of N- and O-glycosylation have been proven to be present in plants [7,44,53], algae and diatoms [54,55], animals [46,56,57,58], fungi [59,60], bacteria [61,62], Archaea [63] and viruses [64]. In the following paragraphs, only the hallmarks of the processes of N- and O-glycosylation in plants are briefly highlighted. For more detail, we refer to some recent review papers about N- and O-glycosylation [7,24,44,49,65,66,67].

### 1.3. Protein Glycosylation

#### 1.3.1. N-Glycosylation

**Figure 2:**
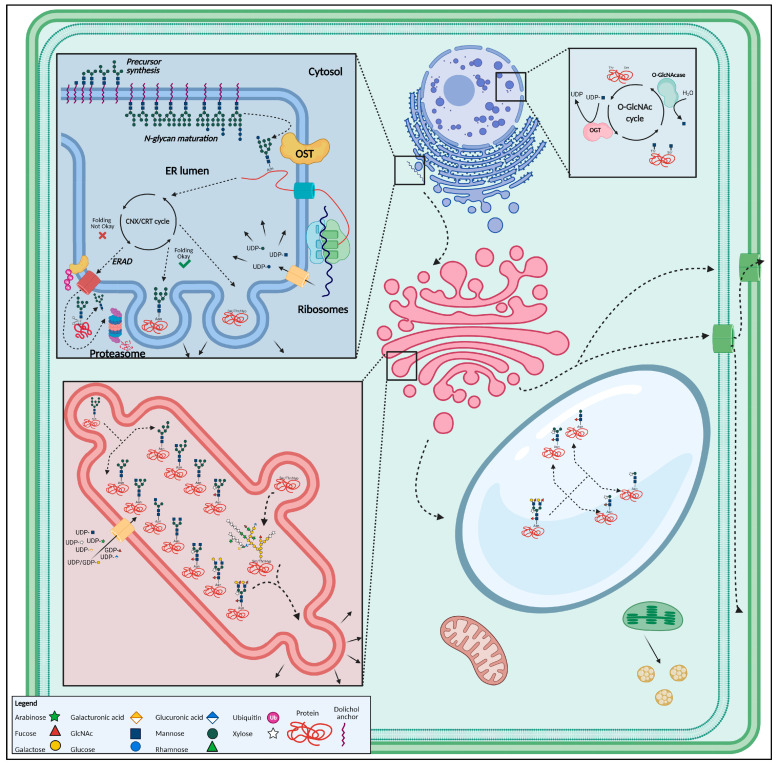
Overview of the most important glycosylated structures in the plant cell. N-glycan synthesis initiates in the endoplasmic reticulum, after which the glycan is added onto a polypeptide. Well-folded proteins are then guided towards the Golgi apparatus through vesicular transport, where the glycan structure is modified. After glycan maturation in the Golgi, glycoproteins are transported towards the vacuole, plasma membrane, cell wall or secreted. O-glycosylation occurs mainly in the Golgi apparatus. The O-GlcNAc modification takes place in the nucleus and cytoplasm. This figure was created with BioRender.com.

N-glycosylation, or asparagine-linked glycosylation is one of the most predominant co- and post-translational modifications that occur during or after protein biosynthesis. The glycan structure is linked to the amide of the asparagine residue, which is always part of the canonical consensus sequence, also called ‘sequon’, Asn-X-Ser/Thr. Here, X is any proteinogenic amino acid but proline, although it has been shown that some plant proteins can be N-glycosylated at a rather unusual, non-canonical Asn-X-Cys site [68,69]. Independent of the canonical nature of the sequon, the N-glycosylation process takes place in the endoplasmic reticulum (ER) and Golgi apparatus and requires the specific action of numerous CAZymes like glycosyltransferases (GTs) and GHs [70]. In plants, N-glycosylation can be seen as an engaged sequence of steps. The N-glycosylation process is pursued as follows (Figure 2):

1. Stepwise assembly of the Glc_3_Man_9_GlcNAc_2_-Dol-PP precursor build-up from Glc, mannose (Man), N-acetylglucosamine (GlcNAc) and a dolichol pyrophosphate (Dol-PP) lipid membrane anchor, in the cytosol and ER lumen [71];
2. En bloc transfer of the N-glycan precursor to synthesized proteins in the ER lumen, through the oligosaccharyltransferase (OST) complex, which involves the actual transfer of the precursor to a protein, co-translationally, thus creating a glycoprotein [72];
3. Processing of the N-glycan and channeling of correctly folded glycoproteins towards the *cis*-side of the Golgi apparatus whereas incorrectly folded glycoproteins, in case re-glycosylation did not yield a properly folded glycoprotein, are directed towards the ER-Associated Degradation (ERAD) pathway [65];
4. Further processing, and modifications and maturation of the N-glycans occurs enzymatically, by the successive action of multiple GTs and GHs, while the glycoprotein is transported from the *cis*-Golgi to the cisternal *trans*-Golgi. Modifications include Man trimming as well as the addition of monosaccharides. Man trimming results in the removal of two to three Man residues from the high-Man N-glycans, creating oligo-Man N-glycans with five (Man_5_) or six (Man_6_) residues [65,73]. The addition of nucleotide-coupled monosaccharides like fucose (Fuc), xylose (Xyl) or GlcNAc to the pentavalent or hexavalent mannose structures gives rise to the typical plant complex and hybrid N-glycans [46,74,75]. However, the hybrid type is rather rare in plants [44];
5. Glycoproteins continue their way along the secretory pathway and end up in the vacuole, plasma membrane or get secreted extracellularly with possible additional modifications, such as vacuolar glycan trimming, giving rise to typical truncated vacuolar (i.e., paucimannosidic) N-glycans [76,77,78,79].

#### 1.3.2. O-Glycosylation

O-glycosylation, or serine/threonine-linked glycosylation, is fundamentally different from N-glycosylation and was less studied for a long time in plants. However, it is receiving the attention it deserves by more intensive studies over the past few years [24,50,80]. In contrast to N-glycosylation, O-glycosylation is a true post-translational modification that does not require a canonical consensus sequence. Plant O-glycosylation is confined to hydroxyproline (Hyp) rich glycoproteins (HRGPs), including moderately glycosylated EXTs, hyperglycosylated arabinogalactan proteins (AGPs) as well as Hyp-rich proteins (HRPs) which are glycosylated in various degrees [24,49,81,82]. Based on their amino acid composition, AGPs may be classified into various subtypes [83]. For a comprehensive overview of AGPs and O-glycan synthesis, we refer to the recent reviews of Seifert [24], Silva et al. [80] and Strasser et al. [7]. The O-glycosylation process takes place in the Golgi apparatus and cytoplasm and, similar to N-glycosylation, requires the specific action of numerous CAZymes [84] (Figure 2). Plant O-glycosylated proteins usually end up in the plant cell wall, although some O-GlcNAcylated or O-fucosylated proteins are also found in the cytosol and the nucleus [85,86,87]. The diversity of O-glycans is huge compared to N-glycans, reflecting the complexity of the O-glycosylation process. While common threads exist in the N-glycosylation process, these are hard to find in the O-glycosylation process. Vertebrates, invertebrates, plants, insects, bacteria and yeasts possess fundamentally different typical O-glycan structures while all N-glycans have a common core [85,88]. The knowledge about animal O-glycosylation, and mammalian O-glycosylation in particular, is very extensive compared to the plant O-glycosylation process [89]. Mucin type O-glycans have not been found in plants. In mosses and vascular plants, other types of O-glycosylation are more common, such as nucleocytoplasmic O-GlcNAcylation, O-GalNAcylation, O-fucosylation, O-linked Gal and oligo-arabinose (Ara) moieties on serine residues of proteins and HRPs [86,87,90,91,92,93,94,95,96].

#### 1.3.3. C-, P- and S-Glycosylation

In contrast to the ubiquitous processes of protein N- and O-glycosylation, C-, P- and S-glycosylation of proteins are rare or non-existing in plants and are generally confined to animals and/or micro-organisms. However, there are some unique examples of proteins with divergent glycosylation patterns in mammals. C-mannosylation occurred on human RNase 2 and interleukin-12 on the typical Trp-X-X-Trp site [97]. S-hexosylation was observed on a cysteine residue of the bacterial protein sublancin 168 [98]. P-glycosylation occurs through phosphorylation of a serine residue, for instance the fungal proteinase I or proteins from protozoan parasites [99]. To date, not one example of these types of protein glycosylation has been reported in plants. However, small molecules can be C-, P- or S-glycosylated in plants [100].

### 1.4. Glycolipids

Lipids comprise a very broad and heterogeneous group of hydrocarbons. In plants, there are a multitude of different lipids such as glycerol, phospholipids, glycolipids and sterols [101]. Lipids are crucial to support life due to their wide variety of roles within the cell, including metabolic, structural and regulatory roles [6,102]. They are the major building blocks of biological membranes that separate the cell from its environment and characterize different subcellular compartments within the cell [6].

Phospholipids, more specifically phosphoglycerolipids, constitute the most abundant group of lipids within the membrane [103]. Glycolipids are defined as molecules that are composed of both a glycan moiety and a lipid moiety [104]. These glycosyl derivatives of lipids can be found throughout the different kingdoms of life: in plants and algae [105], animals [106], bacteria [107] and Archaea [108]. The classification of glycolipids is based on their lipid moieties, including glycoglycerolipids, glycophosphatidylinositols and glycosphingolipids [104]. Additionally, glycosylated derivatives of sterols and fatty acids have been described as well [109,110].

### 1.5. Nucleotide Sugars as Glycan Precursors

The action of GTs is required to assemble glycans by transferring nucleotide sugars (i.e., uridine diphosphate (UDP) or guanidine diphosphate (GDP)-coupled monosaccharides) to proteins. Nucleotide sugars arise through ab initio synthesis, breakdown of putatively misfolded glycoproteins and glycolipids, cell wall reorganizations or pathogenic interactions [111,112,113]. Precursor molecules for nucleotide sugars are Glc-1-phosphate and Man-1-phosphate and originate during photosynthesis [114], or in the dark originating from starch [115]. Starting from precursor molecules, nucleotide sugars are generated in the cytosol through a series of oxidation, epimerization and decarboxylation reactions [116]. Synthesized nucleotide sugars are subsequently transferred to the ER and Golgi apparatus lumen through nucleotide-sugar transporters [111]. These transporters have been identified in various organisms [44]. For instance, in *Arabidopsis* the REPRESSOR OF CYTOKININ DEFICIENCY1 (ROCK1) transporter was identified as an ER-localized UDP-GlcNAc and UDP-GalNAc transporter. ROCK1 function is associated with cytokinin signaling. Mutant *rock1* plants were characterized by an increased organ formation rate, upregulated unfolded protein response (UPR) and phenotypes that suggest a cytokinin shortage [117]. An extensive overview on nucleotide sugar synthesis is given by Decker and Kleczkowski [113].

## 2. Developmental Consequences of Glycosylation: From Flowers to Germinating Seeds

The importance of glycosylation for flowers, fertilization, seeds, developing embryos, germinating seeds and developing roots and leaves is illustrated by the multitude of research articles addressing this topic [7,24,44,49] (Figure 1). Most of these reports make use of mutant plants, mostly in *Arabidopsis thaliana* L., in which one or multiple proteins involved in the glycosylation pathway are knocked out, knocked down or over-expressed, allowing to view which modifications play a crucial role in the glycosylation pipelines.

The following chapters focus on the importance of N- and O-glycosylation for the vital processes that take place from the moment a fertile flower is created till the point that the seed germinates, with special attention to the central position of the cell wall. Defects in these crucial processes result in detrimental consequences for the developing organism, indicating the importance of proper glycosylation, as shown in Table 1.

### 2.1. Flowers Have a Sweet Tooth

**Figure 3:**
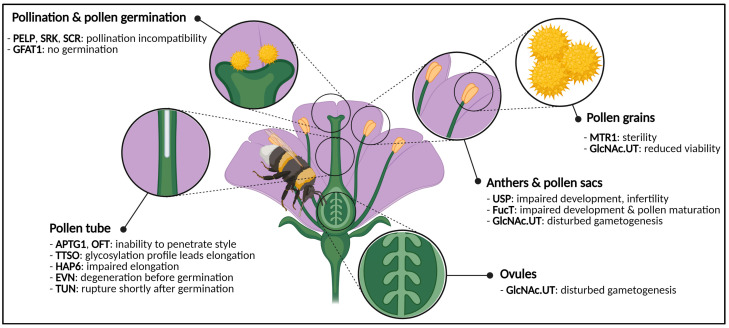
Graphical representation of a generic flower and phenotypes caused by impaired activity of glycosylation enzymes or glycoproteins. Disturbances cause phenotypes in both male and female gametes, anthers, pollen and pollen sacs, pollen germination and pollen tube elongation. Abbreviations: APTG1 (ABNORMAL POLLEN TUBE GUIDANCE1), EVN (EVAN), FucT (α-1,3-fucosyltransferase), GFAT1 (glutamine:fructose-6-phosphate amidotransferase), GlcNAc.UT (GlcNAc-phosphate UDP-transferase), HAP6 (HAPLESS6), MTR1 (MICROSPORE AND TAPETUM REGULATOR1), OFT (O-fucosyltransferase), PELP (Pistil-Specific Extensin-Like Proteins), SCR (S-locus Cysteine Rich), SRK (S-locus Receptor Kinase), TTSO (Transmitting Tissue Specific O-glycoprotein), TUN (TURAN), USP (UDP-sugar pyrophosphorylase). This figure was created with BioRender.com.

Flowers are complex plant organs and exist in all shapes, forms and colors. They are unique to *Angiospermae*, distinguishing them from *Gymnospermae* [170]. After pollination, fruits are formed through enzymatically and hormonally driven processes. While the fruit is developing and is gaining size, shape and volume, plant cells are dividing and elongating, which needs the balanced action of cell wall modifying enzymes. Once the fruit is fully grown, the seed maturation process pursues, the seed gets dispersed and germinates, thereby completing the plants’ developmental cycle. The complexity and diversity of flower morphology reflects the plasticity of all underlying processes [171]. A flowers’ ultimate goal is to produce viable seeds, which will generate progeny and propagate further on. However, flower development is not as straightforward as it seems, since a multitude of requirements have to be met. One of those requirements is proper protein glycosylation as it is responsible for a plethora of developmental processes and even facilitates pivotal events during the plant’s life cycle [150] (Figure 3).

#### 2.1.1. Flower Reproductive Organs Rely on Glycans

Stamens, the male reproductive organs, are comprised of a thin filament and a pollen-containing anther (Figure 3). Male gametes are produced through meiosis-undergoing microspores which develop into pollen. It has been reported that a considerable number of glycoproteins play a crucial role in the development of pollen. These glycoproteins are often leucine-rich repeat (LRR) receptor-like kinases (RLKs) and coordinate cell–cell signaling which is necessary for male reproductivity [172]. Mutants of these glycoproteins often display male sterility. One of several examples is the MICROSPORE AND TAPETUM REGULATOR1 (MTR1) from rice (*O. sativa* L.), a fasciclin N-glycoprotein which is almost exclusively expressed in male reproductive cells. Mutant *mtr1* rice plants showed defects in the tapetum and development of microspores, causing sterility, pointing towards the essential role of proper glycosylation for fertility [173]. Although glycosylation is important for pollen maturation, the process relies on the supply of nucleotide sugars. UDP-sugar-pyrophosphorylase (USP) is involved in the recycling of Ara and Xyl. The effect of impaired nucleotide-sugar recycling for N-glycosylation of pollen-specific glycoproteins was studied in *Arabidopsis*. Knocked out *usp* mutants revealed aberrant anthers and pollen sacks, leading towards infertility [112]. Another UDP-transferase, i.e., a GlcNAc-phosphate UDP-transferase (GlcNAc.UT), which is present in the first step of the hexosamine biosynthetic pathway, is also involved in the development of male flower parts. GlcNAc.UT activity is relevant for GlcNAc delivery during N- and O-glycosylation [149]. Both male and female gametes of *Arabidopsis* plants with the double *glcna.ut1* and *glcna.ut2* mutation were characterized by severe developmental shortcomings. Transmission of gametes was disturbed, due to defects in gametogenesis. Male pollen was less viable, and in some cases female gametogenesis was arrested, leading to an increase in unfertilized ovules. Additionally, the double mutants also displayed embryonal problems, shorter siliques with fewer and shrunken seeds [124]. Furthermore, anther development and pollen maturation also require the action of an N-glycosylation enzyme, i.e., α-1,4-fucosyltransferase (FucT). FucT activity comes to stage during the final steps of the N-glycosylation pipeline when the glycoprotein travels through the Golgi apparatus and receives the Lewis A motif [44,174] (Figure 2). In flowers of tobacco (*Nicotiana tabacum* L.), FucT activity was observed and thought to be developmentally driven. Activity of FucT in stamen was significantly higher compared to other flower parts (i.e., sepals, petals and pistils). Proper α-1,4-fucosylation of pollen-specific glycoproteins seems to be of paramount importance for the developing anthers and pollen [175].

Pistils, the female reproductive organs, comprise the pollen-receiving stigma, the pollen-tube guiding style and ovaries (Figure 3). Recently, it was shown that cell wall O-glycans (i.e., AGPs) play a crucial role during pistil development in tomato flowers during the progamic phase. It was concluded that AGP accumulation correlates with stigma maturation [176]. A comprehensive overview of the role of O-glycans for plant development, and floral development in particular is given by Seifert [24]. Immunocytochemical experiments in flowers of fava bean (*Vicia faba* L.) revealed that AGPs were remarkably present in the stigma and stylar canal [177]. Moreover, it was proven in domesticated apple (*Malus domestica* L.) that AGPs play a crucial role in trafficking the elongating pollen tube towards the ovaries [178]. ABNORMAL POLLEN TUBE GUIDANCE1 (APTG1), an ER enzyme from *Arabidopsis* with mannosyltransferase activity, is of importance during N-glycan precursor synthesis but is also involved in guidance of the pollen tube through the style. Mutant *aptg1* plants were unable to penetrate the style, causing embryo lethality [120].

#### 2.1.2. Sugars: Aphrodisiacs for Plants?

Pollination encompasses the transfer from male pollen and reception by the female stigma. Flowers can get pollinated through self-pollination or cross-pollination, depending on the reproductive organization. As soon as the pollen grain is nested in the stigma and hydrated, the pollen grains germinate and create the pollen tube, which elongates towards the female ovaries [179] (Figure 3). The interaction between male and female gametes, and the involvement of AGPs in particular, has been summarized by Pereira [66] and Seifert [24]. Fertilization encompasses the transfer from male gametes through the pollen tube towards the female gamete in the embryo sac. Once the gametes are merged, the zygote is created, and will develop into an embryo. Simultaneously, the development of fruits (see Section 2.2) and seeds (see Section 2.3) is initiated.

In *Arabidopsis*, pollen grain’s ability to germinate is affected by the action of glutamine:fructose-6-phosphate amidotransferase 1 (GFAT1), a part of the hexosamine biosynthetic pathway. Mutant *gfat1* plants were able to produce pollen, but showed aberrant cell walls with disrupted deposition of pectin and callose, causing male sterility. Furthermore, *gfat1* plants were unable to germinate unless glucosamine was added to the growth medium [148].

The glycosylation profile of a specific Transmitting Tissue Specific O-glycoprotein (TTSO) (i.e., AGP) guides the pollen tube in jasmine tobacco (*N. alata* L.). Moreover, the extent of de-glycosylation was decisive for the pollen tube orientation [180]. Next to AGPs, also extensins are involved in the process of pollination. Pistil-specific EXT-Like Proteins (PELP) make sure that the appropriate pollen type will germinate on compatible stigmas. This form of incompatibility prevents interspecies hybridization [178,181,182]. The concept of incompatibility can also apply to self-pollination. In *Brassicaceae* a stigma-membranal S-locus Receptor Kinase (SRK) interacts with an S-locus Cysteine Rich (SCR) protein from pollen grains, present in the pollen coat. The extent to which SRK and SCR are N-glycosylated decides whether or not they belong to the same haplotype. Only if these two proteins are from different haplotypes (i.e., cross-pollination) pollen tube elongation will be made possible, thereby preventing self-pollination [183].

In some cases, disruption of N-glycosylation-related proteins can jeopardize the plants’ fertility. The HAPLESS (HAP) genes have been identified in *Arabidopsis* and represent a series of genes which are of importance for the development of pollen grains and germination as well as pollen tube elongation [126]. One of the HAP proteins, HAP6, also known as ribophorin 2, is a subunit of the OST which facilitates en bloc transfer of polypeptides from the cytosol to the ER lumen [184] (Figure 2). Mutant *hap6* plants were characterized by shorter pollen tubes which could not exit the style, leading to impaired pollen tube elongation. Other *hap* mutants have been generated and are classified in different phenotypic classes. Each mutant displays aberrant phenotypes that disturb a normal fertilization [126]. O-fucosylation is also a key player during pollination and pollen tube elongation. A Golgi O-fucosyltransferase (OFT) from *Arabidopsis* was discovered. Pollen tubes of mutated *oft1* plants were unable to penetrate the style and to fertilize the ovaries [150]. Even when pollen tube elongation goes without problems, pollen tube perception may be problematic. Pollen tube perception refers to the event in which the male and female gametophytes come in close proximity to each other, and can be mediated by the FERONIA-pathway [185]. TURAN (TUN) and EVAN (EVN) are both part of the FERONIA (FER) (i.e., named after the eponymous glycosylated RLK) pathway and encode a UDP-glycosyltransferase and dolichol kinase, respectively. EVN is related to the N-glycosylation pathway by its involvement in N-glycan precursor synthesis [123]. The involvement of TUN in the N-glycosylation pathway was deduced from RNA interference (RNAi) experiments. Knocked down TUN lines had altered glycosylation profiles of the FER glycoprotein and displayed phenotypes that are similar to the *ost3/6-2* mutants of the OST, suggesting a role in the N-glycosylation pathway at the level of en bloc transfer of the N-glycan precursor [123,127]. Knocked out *tun* and *evn* mutants showed distinct pollen defects. Both *tun* and *evn* mutants failed to generate progeny. Mutant *evn* pollen showed degeneration before pollen germination. Mutant *tun* pollen on the other hand were able to germinate, although the pollen tube ruptured shortly after germination. It was hypothesized that this could be provoked by ERAD of FERONIA-like glycoproteins (i.e., ANX1/2) [123] (Figure 2).

Taken together, both N- and O-glycosylation are of importance during the development of the flowers’ reproductive organs. If glycosylation of glycoproteins fails, flower organs cannot develop properly, gametes end up infertile or fertilization is blocked by pollen tube problems (Figure 3).

### 2.2. Eradication of Sweet Cell Walls Mediates Fruit Ripening

**Figure 4:**
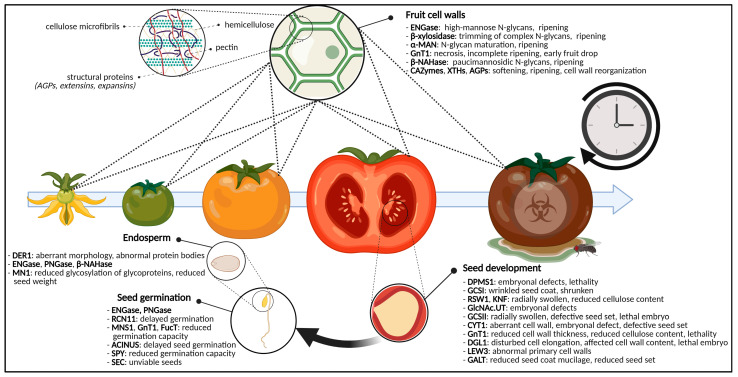
Graphical representation of the glycosylation-related enzymes and their role in fruit ripening and seed development and germination. For fruits, specific glycan-degrading enzymes and CAZymes are important for ripening and softening. Defects in glycosylation enzymes cause aberrant seed morphologies, cell wall shortcomings, embryonal defects, (embryo)lethality and reduced seed set. For the germinating seed, disturbed glycosylation enzymes will postpone germination, reduce germination capacity or will yield unviable seeds. Additionally, the glycosylation state of endosperm glycoproteins will cause certain developmental phenotypes. Abbreviations: ACINUS (Apoptotic Chromatin condensation Inducer in the Nucleus), AGPs (arabinogalactan proteins), α-MAN (α-mannosidase), β-NAHase (β-N-acetylhexosaminidase), CAZymes (carbohydrate-active enzymes), CYT1 (GTP:α-D-mannose-1-phosphate guanylyltransferase), DER1 (DEGRADATION IN THE ENDOPLASMIC RETICULUM1), DGL1 (DEFECTIVE IN GLYCOSYLATION1), DPMS1 (dolichol phosphate mannose synthase complex 1), ENGase (endo-N-acetyl-β-D-glucosaminidase), FucT (α-1,3-fucosyltransferase), GALT (galactosyltransferase), GCSI (α-glucosidase I), GCSII (α-glucosidase II), GlcNAc.UT (GlcNAc-phosphate UDP-transferase), GnT1 (N-acetylglucosaminyltransferase I), KNF (KNOPF), LEW3 (Leaf Wilting 3), MN1 (Miniature1), MNS1 (mannosidase I), PNGase (peptide-N4-(N-acetyl-β-D-glucosaminyl) asparagine amidase), RCN11 (Reduced Culm Number 11), RSW1 (RADIALLY SWOLLEN1), SEC (SECRET AGENT), SPY (SPINDLY), XTH (xyloglucan endotransglycosylase/hydrolase). This figure was created with BioRender.com.

Fruit ripening encompasses the set of processes that facilitate fruit set, development and maturation of fruits (Figure 4). These are well-understood on a biochemical, physical and molecular level, and have been studied thoroughly. Starting from anthesis and fertilization of the ovaries, the seed as well as the surrounding fruit develops through cell division, cell enlargement and maturation [186,187]. Finally, fruits bearing mature seeds will abscise from the parental plant and start their own life cycle. The process of seed development is discussed further on in this review, see Section 2.3.

The ripening process is (epi)genetically, hormonally and environmentally driven, and requires the action of specific pathways that contribute to morphological and sensory properties [187,188,189,190,191]. On a biochemical level, fruit ripening is associated with general softening, caused by enzymatic (i.e., by means of CAZymes) and non-enzymatic (i.e., by means of expansins) cell wall degradation and rearrangement. In addition, also color development, flavor formation and increased pathogen susceptibility coincide with the fruit ripening process [191,192,193,194]. In the next paragraphs, the specific role of glycosylation and glycoproteins in the developmental process of fruit ripening is discussed.

#### 2.2.1. N-Glycans Control Fruit Ripening

Free N-glycans exist in two forms, either the high-Man type or the complex type. They are released from glycoproteins by means of glycanases [78,195]. Endo-N-acetyl-β-D-glucosaminidase (ENGase) and peptide-N^4^-(N-acetyl-β-D-glucosaminyl) asparagine amidase (PNGase) give rise to high-Man and complex glycans, respectively, although ENGase exerts the biggest influences on fruit ripening [78,137,196,197,198]. Additionally, β-xylosidase, residing in fruits, has been reported to cleave off glycans from complex-type N-glycans [199]. The N-glycanase activity is also important as part of the ERAD quality control [44,200] (Figure 2).

The presence of unconjugated N-glycans in ripening fruit pericarp was mentioned by Priem et al. [201]. It was observed that the amount of free N-glycans significantly increased during the ripening process of tomato (*Solanum lycopersicum* L.) fruits. Treating tomato pericarp with different free N-glycans caused either a delay or a stimulation in fruit ripening [202]. Treatment with tunicamycin (i.e., a glycosylation inhibitor) prevented the ripening process [203,204]. Furthermore, the activity and gene expression of ENGase as well as the content of free N-glycans was measured during the ripening [197]. It was shown that although ENGase expression and activity remained equal, the amount of high-Man N-glycans increased during the late ripening stages. The high-Man and truncated complex type N-glycans accounted for, respectively, 22% and 70% of the total amount of N-glycans in ripe tomato fruits [205].

More proof of glycan maturation and the role of free N-glycans in fruit ripening was reported for bell pepper (*Capsicum annuum* L.) and woodland strawberry (*Fragaria ananassa* Duchesne). Two N-glycan processing CAZymes, α-mannosidase (α-MAN) and β-D-N-acetylhexosaminidase (β-NAHase), play a role in fruit ripening [138,139,206,207]. Both enzymes are active on N-glycans from the cell wall. α-MAN is involved in cell wall glycan trimming and β-NAHase creates paucimannosidic N-glycans by removing terminal GlcNAc or GalNAc residues [208] (Figure 2). Their activity and expression levels in bell pepper were proven to increase during fruit ripening. RNAi showed that fruit softening was significantly reduced and deterioration could be delayed up to 7 days [139,209]. Recently, the RIPENING INHIBITOR (RIN) transcription factor regulating α-MAN transcription during tomato fruit ripening was discovered. In knocked out *rin* mutants, α-MAN activity was suppressed. However, the activity was partially restored by treating *rin* mutants with 1-aminocyclopropane-1-carboxylic acid, a precursor of ethylene biosynthesis, the ripening hormone, suggesting a significant interplay between the RIN transcription factor and ethylene signaling [206]. Additionally, it was also demonstrated by RNAi experiments that the reduction in N-acetylglucosaminyl transferase I activity (GnT1) in tomatoes causes the appearance of necrosis as well as incomplete ripening combined with early fruit drop and increased susceptibility towards pathogens. Interestingly, when Golgi α-mannosidase (MANII) activity was also suppressed, the fruits developed normally without necrosis, but contained no or a small number of seeds [143]. The results illustrate the importance of de-N-glycosylation activity and glycan maturation for structural changes during fruit ripening.

#### 2.2.2. Glycoproteins, Polysaccharides and Fruit Ripening

Glycoproteins are abundant in fruits and are differentially expressed along the ripening process (Figure 4). For instance, in tomato fruits, 553 N-glycosites on 363 N-glycoproteins were discovered, of which 191 were differentially expressed between the initial and final stages of fruit ripening. A large share of the differentially expressed glycoproteins can be attributed to starch, Suc and galactose (Gal) metabolism [194].

A particular glycoprotein whose activity is dependent on its glycosylation state is the xyloglucan endotransglycosylase/hydrolase (XTH) from strawberry [210]. Enzymes belonging to the XTH family are involved in the metabolism of xyloglucan, one of the main hemicellulose polysaccharides in the cell wall. The mode of action of XTHs is well-comprehended [211,212,213]. XTHs can accommodate both xyloglucan transglucosylase (XET) and xyloglucan hydrolase (XEH) activities, corresponding with assembly and disassembly of cell wall polysaccharides, respectively. In the plant cell wall, XET activity cleaves xyloglucans from xyloglucan chains and transfers it to a second xyloglucan, thereby integrating new xyloglucans into the cell wall, on the one hand. On the other hand, XTH can also deploy its hydrolase activity, by which xyloglucans are solely cleaved into xyloglucan oligomers without being transferred to other polysaccharides [210,214]. It is reported that XTHs from tomato and persimmon (*Diospyros kaki* Thunb.) are directly involved in ripening and softening of fruits [215,216]. Treating strawberry XTH1 with a PNGase caused a reduction in protein stability and less favorable enzymatic characteristics, highlighting the importance of proper glycosylation of proteins for their activity [210].

Another intriguing class of proteins are the polygalacturonase inhibiting proteins (PGIPs). These are glycoproteins with a function in defence against pathogens by reversibly blocking phytopathogenic secreted polygalacturonases, which could digest pectin from the cell wall and henceforth facilitate pathogenic (i.e., bacteria, fungi, nematodes, parasitic plants) attack [217,218,219,220]. PGIPs are produced along the development of fruits as a result of several biotic and abiotic stresses, and are hypothesized to be present in the cell walls, where they contribute to pectin metabolism [219,221]. PGIPs have a LRR structure and play an important role in tissue development and interaction with pathogens [222]. The extent of PGIP glycosylation varies between species [219]. In common pear (*Pyrus communis* L.), seven glycosites are involved in the specific interaction between PGIPs and polygalacturonases [223].

O-glycosylated AGPs reside in the cell walls and plasma membranes of fruits, and are covalently linked to pectin and xylan. In tomato, non-classical subtype AGPs are constitutively expressed during fruit ripening as well as in response to abiotic stress [224]. Recently, a lot of attention has been paid to AGPs and their involvement in apple fruit ripening [225]. It was shown that AGPs are spatio-temporarily related to fruit ripening and correlated with cell wall reorganization, according to immunolocalization studies [226,227].

However, it was hypothesized that both glycosylation and activity of CAZymes are decisive for fruit ripening [207]. During the ripening process, the cell wall undergoes significant structural changes in cellulose, hemicellulose and pectin content [228]. Polymers from cell walls and cell wall matrix are depolymerized, solubilized and rearranged, although different fruits can ripen in different ways and not all polymers in different fruits are affected in the same way. Furthermore, not only polymers but also AGPs are degraded during fruit ripening [193]. The cell wall degrading activity of various CAZymes such as exo- and endo-polygalacturonase, pectin methylesterase, pectate lyase, endo-1,4-β-glucanase, endo-1,4-β-mannanase, α-arabinosidase and β-galactosidase was reported in various fruits [193,207,229,230,231].

### 2.3. The Sugar-Craving Cell Wall of Seeds

#### 2.3.1. The Ever-Changing Cell Wall

Plant cell walls are complex and dynamic organs which fulfil many essential functions. Cell walls act as cellular exoskeletons and give shape, volume, structure and a certain degree of rigidity and strength to plant cells. Their rate and ability to expand is mainly dictated by its rigidity, which is determined by its composition [232]. During the development of the cell, the cell wall changes in shape in order to allow cellular extension and growth. For instance, cell wall disassembly is necessary in the endosperm of germinating seeds, but also during fruit ripening and leaf abscission. Next to structural traits and involvement in plant development, cell walls are also involved in intracellular communication and defense against biotic and abiotic stresses [233,234].

Cell walls are built up of O-glycosidic biomolecules, grouped in cellulose, pectic polysaccharides, hemicellulose polysaccharides and lignin. Pectin and lignin are unique to the primary cell wall and secondary cell wall, respectively, and the specific composition of the cell wall varies between different plant species [235]. The cell plate is formed at the end of cytokinesis through phragmoplast-assisted assembly. The latter is described as a scaffold of microtubules and actin filaments which guide Golgi vesicles to deposit pectin, hemicellulose polysaccharides as well as AGPs, the building blocks of the cell plate [236]. The cell plate gives rise to the plasma membrane as well as the middle lamella. Due to pectin deposition and interweaving, the middle lamella is formed. The middle lamella fixates neighboring cells and keeps them from moving, which is in contrast to growing animal cells [237]. After the formation of the middle lamella, the protoplasts of the two daughter cells deposit microfibrils, which are precursors to adhere the primary cell wall. Whenever the cell is fully grown, a specialized secondary cell wall is layered on top of the primary cell wall and is connected with it by means of intertwined microfibrils [238].

#### 2.3.2. Cellulose Biosynthesis-Related Problems Cause Cell Wall Disruptions

Two classical examples of enzymes that are of great importance for both plant N-glycosylation and seed and embryonal development are the trivalent dolichol phosphate mannose synthase (DPMS) complex [122] and α-glucosidase I (GCSI) [131]. Although DPMS and GCSI are involved in very different steps of the glycosylation pathway, plants display comparable phenotypes whenever the coding gene is interrupted or over-expressed. Whereas the DPMS complex plays a pivotal role in the synthesis of the lipid-linked glycan precursor [71], α-glucosidase activity is required during glycan trimming after en bloc transfer of the N-glycan precursor [130] (Figure 2). Both over-expressed DPMS1 and knocked out *gcsI* mutants display a lethal phenotype with detrimental defects on embryonal development. One of the phenotypic particularities shared between over-expressed *DPMS1* plants and mutant *gcs1* plants is the aberrant seed morphology: either wrinkled or shrunken, or both. In both cases, the aberrant phenotypes can be attributed to disturbances in seed coat cell wall formation and cellulose biosynthesis [122,130,131]. Shrunken seeds with defects in the embryonal stage were also observed when two *GlcNA.UT* genes were knocked down in *Arabidopsis*. However, the observed phenotype was not linked to cell wall defects [124]. Other α-glucosidase-encoding genes are Radially Swollen (RSW) 1 and KNOPF (KNF) and both are involved in cellulose biosynthesis and morphogenesis during embryonal development. Embryos of *rsw3* and *knf14* mutants showed serious defects in embryogenesis, looked radially swollen and were highly reduced in cellulose content [131]. The observed phenotype was attributed to the UPR. These findings suggest that N-glycosylation is of importance for cell wall formation through cellulose biosynthesis. Additionally, *gcsII* mutants, also involved in glycan trimming downstream of GCSI, have been reported and linked to cellulose biosynthesis. These mutants also displayed radial swelling of the roots, lethal embryos and defects in seed setting [132,133]. Very comparable phenotypes have been observed in mutants of the GTP:α-D-mannose-1-phosphate guanylyltransferase (CYT1) enzyme in *Arabidopsis* which is, just like DPMS1, involved in N-glycan precursor synthesis by producing GDP-Man and GDP-Fuc (Figure 2). Distorted cell walls, disturbed embryonal development and lethality have been reported for *cyt1* mutants [121]. Both GCSI and GCSII activity, and N-glycan precursor synthesis are important for cell wall construction through cellulose biosynthesis [131,239].

An additional example to illustrate the link between glycosylation, embryonal development and cell wall synthesis is the role of GnT1. GnT1 activity is required for glycan maturation in the Golgi apparatus and is needed for normal plant development in rice. Phenotypes of *gnt1* mutants were characterized with severe cell wall shortcomings. Cell wall synthesis was incomplete, leading to reduced thickness and cellulose content. Eventually, the *gnt1* mutant caused lethality without reaching the reproductive stage [142]. Another lethal mutant was also observed in *Arabidopsis* when the DEFECTIVE IN GLYCOSYLATION 1 (DGL1) gene, a subunit of the OST, was knocked out. The *dgl1-1* mutant revealed a reduction in N-glycosylation of a protein disulfide isomerase, an ER glycoprotein involved in properly processing ER-residing proteins [125,240]. Furthermore, the *dgl1-1* mutant showed defects in cell elongation and an altered non-cellulosic polysaccharide composition, but no cellulose deficiencies. In addition, an even more severe *dgl1-2* mutant was obtained which was embryo-lethal. The obtained phenotypes support the idea that glycosylation is important for the developing plant [125].

Leaf Wilting 3 (LEW3), encoding an α-1,2-mannosyltransferase (ALG11) necessary for N-glycan precursor synthesis, plays a role in proper cell wall synthesis in *Arabidopsis*. Mutant *lew3* were characterized by aberrant levels of normal high-Man glycans (i.e., low amounts) and complex glycans (i.e., high amounts). Furthermore, the *lew3* mutants displayed abnormal plant development, impaired fertility and reduced cellulose synthesis, causing abnormalities in the primary cell wall. Moreover, the sensitivity of *lew3* mutants for ABA, and osmotic stress was altered [119]. These results indicate that N-glycosylation plays a role in embryonal development, but also affects sensitivity towards ABA, which negatively influences germination [14].

Beihammer et al. [241] recently reported that a considerable number of glycoproteins originating from *Arabidopsis*, tobacco (*N. benthamiana* L.) and rice, decorated with the Lewis A motif, are heavily involved in the synthesis of primary and secondary cell wall constituents. However, when the addition of the Lewis A motif was hindered, no significant changes in cell wall composition were observed [241].

Similar to N-glycosylation, O-glycosylation is involved in proper cell wall formation in seeds. AGPs play a crucial role in the development of flowering plants [24,49,66]. In general, the contribution of O-Hyp glycans is also situated at the level of the cell wall, where they make up one third of the total protein content. The extent to which AGPs are involved in plant development has already been discussed in detail by several authors [24,49,66]. Most of the research encompasses discoveries concerning the reproductive plant and fertility. Less is known and studied about the relationship between O-glycans, modulation of the cell wall in connection to seed development. However, some examples can be found in the literature. The multigene family of galactosyltransferases (GALTs) from *Arabidopsis* is represented by five members and facilitates the initial step of galactosylation of a fasciclin-like AGP named Salt-overly Sensitive (SOS) 5. Most fasciclins contain a GPI-anchor and are involved in cell wall biosynthesis [81]. All AtGALTs are expressed in the seed coat during different stages of embryogenesis. Knocked out *galt2* and *galt5* mutants showed reduced seed coat mucilage, while *galt4* and *galt6* revealed a reduced seed set, pointing towards a probable function during seed development [168,169,242].

### 2.4. Glycosylation Decides over Seed Germination

The previously mentioned mutations displayed aberrant phenotypes that disturbed development of the embryo and/or seeds and often resulted in lethality [121,122,130,131] (Table 1). Moreover, these mutations can also affect the germination stage [130] (Figure 4). In germinating rice seeds, the number of N-linked glycosites equaled 242, distributed over 191 glycoproteins, highlighting the abundance and importance of (N-linked) glycosylation for the germinating seed [48]. Results of Horiuchi et al. [243] indicate that the glycosylation pattern of ungerminated and germinated rice seeds changes. Whereas ungerminated rice seeds are characterized by mostly high-Man type N-glycans (16%) and paucimannosidic N-glycans (76.7%), germinated rice seeds show more complex N-glycans with the Lewis A epitope [243]. The following section will highlight some examples of glycosylation-related proteins or enzymes, but also some small molecules, whose function is indispensable for seed germination.

#### 2.4.1. (De)Glycosylation and Seed Germination

ENGase and PNGase are two enzymes with de-N-glycosylation activity [137,196]. Both the activities of ENGase and PNGase were upregulated during seed germination. In barley (*Hordeum vulgare* L. cv. Plaisant) seeds, PNGase activity was almost completely restricted to the endosperm, which gets broken down during germination, while ENGase activity was highest in the developing embryo and seedling [137]. In rice, the highest PNGase activity was measured in the seedlings [196]. The enhanced activity of N-glycanases during germination suggests that de-N-glycosylation is of importance during the early stages of seedling development on one hand, and de-N-glycosylation of endosperm storage glycoproteins during germination on the other hand. The action of N-glycanases releases free N-glycans that can act as growth factors, as demonstrated in flax seedlings [244]. Moreover, free N-glycans play an important role in fruit ripening and adaptation to environmental changes [78].

UDP-glucose:glycoprotein glucosyltransferase (UGGT), is involved in ERAD but also in brassinosteroid signal transduction and can also perform glycosylation [135,245]. The Endoplasmic Reticulum Quality Control (ERQC) or ERAD’s main function is to provide and ensure correctly folded proteins by degrading misfolded proteins [246]. ERQC/ERAD relies on UGGT, which distinguishes misfolded proteins from well-folded proteins (Figure 2). UGGT was discovered during research regarding the BRASSINOSTEROID INSENSITIVE1 (BRI1) receptor, perceivers of brassinosteroids, which play crucial roles in modulating seed germination, plant growth and differentiation [247,248,249]. Mutant *bri1* plants are characterized by a series of phenotypes and cause the mutated receptor to end up in ERAD. For instance, *bri1-4* plants rarely produce seeds [250]. Interrupted UGGT activity prevents *bri1* mutants to be re-glycosylated, which withdraws them from ERAD. The defects resulting from the lack or disturbance of BRI1 or UGGT activity highlight the importance of brassinosteroid signaling and proper ERAD functioning for the production and germination of seeds.

#### 2.4.2. Glycan Maturation in the Golgi Apparatus Affects Seed Germination

Glycan maturation takes place mainly in the Golgi-apparatus and relies on the action of multiple enzymes (Figure 2). β-1,2-xylosyltransferase (XylT) activity is important for proper glycan maturation and seed germination. XylT activity is required for the transfer of Xyl from UDP-Xyl to the core Man-unit of N-glycans during the glycan’s passage through the Golgi apparatus [146,251]. In rice, XylT activity is encoded by the Reduced Culm Number 11 (RCN11) gene [147]. Additionally, the rice RCN11 protein is involved in mediating root development under anoxic conditions and ABA signaling during seed germination. Mutant *rcn11* plants were lacking XylT activity, causing a series of effects. For instance, it was observed that *rcn11* rice seeds treated with various concentrations of ABA, germinated significantly later compared to wild type rice seeds, suggesting a role for RCN11 in sensing ABA. The findings of Takano et al. [147] reveal that XylT activity is important and that particular unidentified glycoproteins with β-1,2-Xyl residues plays an essential role in seed germination.

The role of various Golgi GTs was investigated in Japanese trefoil (*Lotus japonicus* L.). A collection of LORE1 N-glycan maturation insertion mutants was generated and resulted in modified glycans of glycoproteins, such as convicilin 2. Interruption of the Golgi GTs MNS1 (mannosidase I), GnT1 and FucT had serious consequences for seed production and plant growth. However, seed germination was not altered. Disturbance of the LjGnT1 gene yielded the most severe effect on the growth of Japanese trefoil plants, which died before reaching the flowering phase. Surviving plants reached the reproductive stage, but produced less seeds, highlighting the importance of glycan maturation for the production of seeds and plant growth [140].

All mentioned examples illustrate how the presence or absence of particular N- and O-glycosylation-related enzymes have detrimental consequences for the developing plant at the level of the developing embryo and the germinating seed (Table 1). In the case of ALG11, CYT1, DPMS1, GCSI, GCSII and GnT1, all but one ER enzymes, mutations had clear implications on the developing embryo at the level of the cell wall [119,121,122,130,131,132,133,142]. For MNS1, XylT, FucT, Golgi enzymes, mutations also have implications on the germination capacity of the seed [140]. Furthermore, ERAD-related enzymes like PNGase and UGGT exert an impact on the germination capacity of seeds [135,136,137]. All these conclusions point out and confirm that proper N- and O-glycosylation are of paramount importance for plant development.

#### 2.4.3. O-GlcNAc Modification on Cytosolic Proteins Is Important for Seed Germination

O-glycosylation in the cytoplasm has also been reported to be involved in seed germination. More specifically, a cytosolic O-fucosyltransferase (OFT) named SPINDLY (SPY), involved in the signaling of GAs and other developmental aspects, was reported in many plants, including *Arabidopsis*, *Petunia*, tomato, rice and barley [152,252,253,254]. Furthermore, the link between SPY and influence on germination capacity was shown. Mutant *spy-4* seeds were able to germinate in the presence of paclobutrazol, a germination inhibitor. Singh et al. [255] concluded that SPY negatively regulates GA response, and therefore also influences germination of seeds. Interestingly, *spy* mutants are also able to suppress several other severe developmental defects when combined with other genes. In *Arabidopsis*, APETALA2 (AP2) is involved in fruit development [256]. The *spy-ap2* mutant showed suppression of auxiliary flower production and floral branching [257]. More flower-related defects were reported when the GA-synthesizing gene from pea (*Pisum sativum* L.), GA 2-Oxidase2 (2OX2), was knocked out and combined with *spy*. The *spy-2ox2* mutant showed suppression of pollen tube growth and elongation defects [255]. The significance of the relation between O-GlcNAcylation and plant development, combined with signaling of GAs, was illustrated when SECRET AGENT (SEC), an O-GlcNAc transferase (OGT) from *Arabidopsis*, was knocked out in combination with *spy*. Mutant *spy-sec* plants carried inviable seeds [90,151,258].

A recent example of an O-GlcNAcylated and O-fucosylated glycoprotein is AtACINUS, the *Arabidopsis* homolog of the mammalian Apoptotic Chromatin condensation Inducer in the Nucleus. It was shown that knocking out *AtACINUS* and a paralog (i.e., *AtPININ*) gave rise to aberrant developmental phenotypes such as dwarfism, delayed seed germination and flowering, and an increased sensitivity towards ABA. It was illustrated that the function of AtACINUS relies on its O-glycosylation state, and that O-glycosylation is important for the regulation of alternative splicing [86,259].

#### 2.4.4. Glycosylation of Endosperm Glycoproteins Influences Seed Morphology and Germination

Next to large amounts of reserve sugars stored as starch, the seed endosperm also contains a considerable amount of storage proteins, which act as an amino acid warehouse [260] (Figure 4). Storage proteins can be found throughout the whole plant, in seeds, roots, shoot tubers, wood and bark [261]. Storage proteins are synthesized in the ER and get processed through the endomembrane system [261,262]. Correctly modified and folded storage protein can be deposited in ER-residing protein bodies, or can be transferred to protein storage vacuoles after passage through the Golgi apparatus (Figure 2). Next paragraphs focus on examples of endosperm-related glycoproteins and their role for seed development and germination.

Maize storage proteins, called zeins, comprise mostly prolamins and small amounts of globulins. Localization studies in germinating maize endosperm revealed that endosperm-related glycoproteins undergo a shift in localization and N-glycan profile during seed maturation. Compared to fresh non-matured maize seeds, mid-maturation seeds showed significantly higher numbers of protein bodies. In addition, protein storage vacuoles were more abundant. A notable shift in subcellular localization was observed for phytase, a maize glycoprotein, during seed maturation. In fresh and mid-maturation seeds, phytase is decorated with paucimannosidic N-glycans, which corresponds with the vacuolar location [263]. When the seed reached maturity, phytase was provided with trimmed N-glycans without fucose and was localized to ER-related protein bodies [264].

Specific to the endosperm of rice seeds, a new DEGRADATION IN THE ENDOPLASMIC RETICULUM1 (DER1) homolog which interacts with the ERAD pathway was reported. Confirmed by co-immunoprecipitation, OsDER1 proved to interact with parts of the yeast-homolog of the E3 ubiquitin ligase complex [265]. The latter is a retro-translocation channel which directs misfolded polypeptides through the ER-membrane [266]. Overexpression of *OsDER1* resulted in the up-regulation of the unfolded protein response and shrunken seeds. Knocked out *der1* mutants were characterized by abnormal protein bodies in the ER. Furthermore, similar to the plants with overexpressed *DER1*, mutant *der1* seeds had an aberrant morphology. Seeds were longer and thinner compared to the wild-type controls. Moreover, they were opaque, floury and shrunken [267]. The protein bodies looked veined and cracked. The aforementioned phenotypes suggest that OsDER1 is indeed involved in ERAD and impacts the household of the endosperm-residing storage proteins as well as seed morphology [267].

De-N-glycosylation of endosperm-related glycoproteins has been reported many times before, as discussed earlier in this review. Barley PNGase-activity was predominantly (for 90%) localized to the endosperm [137]. Additionally, in lupin (*Lupinus albus* L.) cotyledons, β-NAHase activity was observed [268]. In plants, β-NAHases are involved in the creation of paucimannosidic N-glycans by the removal of GlcNAc moieties. The activity of β-NAHase is suggested to be of significance during fruit formation and defense against pathogenic fungi [139,269]. Moreover, β-NAHase trims oligosaccharides from storage proteins during germination. De-N-glycosylation pursues simultaneously with mobilization of storage proteins during seed development and seedling growth. Free sugars are then directed to the growing seedling [260,270]. Impairment of de-N-glycosylation activity could hamper seedling development and growth. This idea was confirmed by Silva-Sanchez et al. [271] when a cell wall invertase (INV) from maize endosperm was knocked out, giving rise to the *miniature 1* (*mn1*) phenotype. The involved cell wall INV is responsible for hydrolyzing Suc, which gets depleted by the developing seedling but also serves in sugar signaling [272]. Mutant *mn1* plants showed a significant decrease in glycosylation of endosperm cell wall glycoproteins, a decrease in free monosaccharide content and were characterized by a reduced seed weight (up to 70%) compared to wild-type plants [271,273]. Loss of function of the pertinent cell wall INV did not evoke lethality [274].

#### 2.4.5. Glycosylated Small Molecules and Their Role in Germination

Small molecules are organic compounds which confer a certain biological activity, such as secondary metabolites and hormones [275,276]. Several GTs are involved in the N- and/or O-glycosylation of plant hormones and secondary metabolites. Furthermore, there exists a significant relationship between glycosylation of small molecules and germination.

A first example in *Arabidopsis* which highlights the importance of both N- and O-glycosylation for the germination of seeds is the glycosylation of nicotinate, an intermediate of the NAD salvage pathway [277]. However, O-glycosylation of nicotinate is solely found in *Brassicaceae*. One of the GTs involved in the glycosylation of nicotinate is UDP-GT74F2. The mutant *ugt74f2* knock out line, without glycosylation of nicotinate, was characterized by delayed germination when exposed to abiotic stress. This effect was attributed to the disturbed nicotinate homeostasis caused by the mutation [277].

In addition, many other glycosylated small molecules may claim their function in germination of seeds. UDP-GT74E2 transfers Glc moieties to auxin, involved in modulating the plants’ architecture when exposed to abiotic stress. Recombinant overexpression of *Arabidopsis UGT74E2* in rice drastically altered the hormonal household, but also showed enhanced levels of germination [278]. Glycosylation of plant hormones exerts also a plethora of other functions for plant development and dealing with biotic and abiotic stress. For an overview of glycosylated small molecules, we refer to Behr et al. [279].

Plant sterols can also be glycosylated. It was shown that the sterol glycoside transferase from ashwagandha (*Withania somnifera* L.) caused late germination and stunted growth of tobacco (*N. tabacum* L.) plants. Enhanced expression of *WtSGTL1* in tobacco plants was also associated with the accumulation of glycosylated sterols and increased resistance towards the *Spodoptera litura* moth [280].

## 3. Glycoproteins during Root and Leaf Development

### 3.1. The Deal with Glycosylation in Roots

The root system provides the plant access to nutrients and water. Additionally, the roots serve as interaction site to encounter both symbiotic organisms and pathogens [281].

#### 3.1.1. N-Glycans: Influencers of Root Development

**Figure 5:**
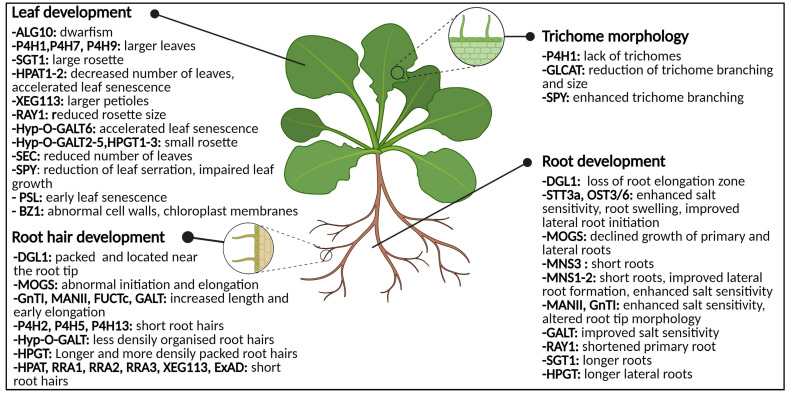
Graphical representation of the glycosylation-related enzymes and their role in leaf development, trichome morphology and root (hair) development. Both abnormal N-glycosylation and O-glycosylation result in changes of phenotype and morphology. Abbreviations: ALG10 (α-1,2-glucosyltransferase 10), BZ1 (Brittle stem and Zebra Leaf 1), DGL1 (DEFECTIVE IN GLYCSOYLATION1), ExAD (extensin arabinose deficient transferase), FUCTc (α1,4-fucosyltransferase), GALT (β1,3-galactosyl-transferase), GLCAT (β-glucuronosyltransferases), GnT1 (N-acetylglucosaminyltransferase I), HPAT1-2 (Hyp O-arabinosyltransferases 1 and 2), HPGT (Hydroxyproline O-galactosyltransferase), Hyp-O-GALT (hydroxyproline-O-β-galactosyltransferase), MANII (α-mannosidase II), MNS (α-mannosidase I), MOGS (mannosyloligosaccharide glucosidase), OST3/6 (oligosaccharyltransferase subunit 3/6), P4H (prolyl 4-hydroxylase), PSL (premature senescence leaf), RAY1 (REDUCED ARABINOSE YARIV1), RRA (REDUCED RESIDUAL ARABINOSE), SEC (SECRET AGENT), SGT (serine O-α-galactosyltransferase), SPY (SPINDLY), STT3a (STAUROSPORIN AND TEMPERATURE SENSITIVE3A), XEG113 (Xylo-endoglucanase113). This figure was created with BioRender.com.

N-glycosylation of different proteins is important for root development. The OST complex, necessary for transfer of the oligosaccharide from the lipid-linked oligosaccharide to a nascent polypeptide chain, consists of multiple subunits and mutations of some of these subunits disturb root growth (Figure 1, Figure 2 and Figure 5) [44]. In both *Arabidopsis* and rice, mutation of the *DGL1* gene resulted in aberrant root phenotypes [125,282]. The *Atdgl1-1* mutant was incapable to develop beyond the post-embryonic phase. Both hypocotyls and roots display considerably reduced growth due to decreased cell elongation. Moreover, in the absence of the elongation zone, root hairs were packed and located close to the root tip [125]. The leaky rice mutant *Osdgl1* exhibited a similar phenotype as *Atdgl1*-1 [282]. Both the *Arabidopsis* and the rice mutant revealed an abnormal composition of the non-cellulosic cell wall polysaccharides [125,282]. Mutations of other subunits in the OST complex did not show any obvious aberrant developmental or morphological root phenotype under normal growth conditions. However, the addition of salt to the growth medium of the mutant lines *staurosporin and temperature sensitive3a* (*stt3a*) and *ost3/6* yielded a decreased root growth in *Arabidopsis* [127,128,129]. Salt stress/osmotic stress induced root swelling and enhanced lateral root initiation in both *stt3a* and *ost3/6* seedlings. In general, under-glycosylation increases osmotic and salt sensitivity [119,127,128,129].

Numerous examples of plants mutated in processing enzymes involved in the maturation of N-glycans displayed defective root growth and root architecture due to aberrant protein N-glycosylation [134,141,145,283] (Figure 5). Mutation of the GCSI ortholog in rice, called mannosyloligosaccharide glucosidase (MOGS), yielded severely disrupted root growth. Both the primary root and the lateral roots revealed reduced growth compared to the wild type plants. Moreover, root hair development was impaired, in particular the initiation and elongation of the root hairs were abnormal [134].

Man trimming defects during N-glycan maturation severely influence root phenotype [283]. MNS1 activity is required in the early processing of the N-glycan moieties of glycoproteins. Three different enzymes were identified in *Arabidopsis* [145,283,284]. The single mutants of *mns1* or *mns2* did not show any aberrant phenotype. The *mns3* mutant and the double mutant *mns1*/*mns2* displayed shorter roots. Moreover, the double mutant had increased lateral root formation. The physiological effects after mutation of all three MNS genes resulted in aberrant cell walls and showed short and swollen roots [145]. Recently, it was reported that particular Man residues in the N-glycan structure are more important as compared to others [283,284]. The hypothesis was confirmed by rescuing the triple *mns* mutant from defective root growth through the elimination of Man residues by mutating ALG genes responsible for the assembly of the oligosaccharide precursor. These data suggest that specific assemblies of oligosaccharides are well tolerated in plants despite being abnormal compared to the wild type N-glycans [283,284].

A recent study highlighted the role of several N-glycan modification enzymes for root architecture in *Arabidopsis* [141]. Aberrant processing of complex N-glycan moieties in mutants *gntI*, *manII*, *galt* and *fuctc* were related to the maturation of the N-glycans present on the glycoproteins affected the root hair system. Mutations resulted in an approximate two-fold increase in the root hair length and an earlier start of root hair elongation. The authors postulate that these abnormal effects originate from the adverse N-glycan modification and the subsequent glycoprotein stability, protein interaction, enzyme kinetics and/or the substrate binding. Moreover, it was suggested that the root hair development was altered due to changes in plant hormone signaling and homeostasis [141,281].

Similar to the mutation of the OST complex subunits, STT3a (STAUROSPORIN AND TEMPERATURE SENSITIVE 3a) and OST3/6, some mutants of genes involved in N-glycan maturation and processing were also affected by abiotic stresses. For instance, in *Arabidopsis* the double mutant *mns1 mns2*, GnT1/complex glycan-less1 (*gntI*/*cgl1*) and α-mannosidase II/hybrid glycosylation 1 (*manII*/*hgl1*) mutants were salt/osmotic stress sensitive [75,144,284]. Salt stress inhibited growth and caused abnormal root-tip morphology in these mutants. The mutant *cgl1* lacked the proper processing of N-glycans due to a defect of the GnT1 gene [144]. Impairment of N-glycosylation in different mutants resulted in perturbations of cellulose biosynthesis or alterations in other cell wall polymers [125,142,145,282]. Maintenance of the cellulose biosynthesis is vital for plants to adapt to external stresses such as salt stress [285]. Recent studies suggested a clear link between salt sensitivity, cellulose biosynthesis and N-glycan maturation in roots [283,286].

However, root phenotypes are not always linked to the lack of glycosylation, but are rather the result of the enhanced UPR and the ER protein quality control pathway due to the accumulation of misfolded proteins [44,67,287,288]. It is noteworthy that there are few studies elucidating the effect of the presence of the glycans on individual glycoproteins. KORRIGAN1/RADIALLY SWOLLEN2 (KOR1/RSW2) is one of these examples. The functionality of the β-1,4-endoglucanase KOR1 is dependent on the presence of the N-glycans [289]. KOR1 contains 8 N-glycosylation sites. The lack of multiple glycans on KOR1 affected subcellular localization and could not support root growth, suggesting that the N-glycan decoration of KOR1 is essential for proper root growth [286,290].

#### 3.1.2. Impaired O-Glycosylation Affects the Root Tips and Root Hairs

Similar to N-glycosylation, O-glycosylation also interferes in root growth (Figure 5). O-glycans determine the role and molecular properties of the HRGP and of small hormone peptides [7]. Prior to the addition of a sugar moiety, modifying the proline to Hyp residue is essential. The generation of Hyp residues is regulated by prolyl 4-hydroxylase (P4H) [291]. Mutational analysis of several P4Hs (P4H2, P4H5, and P4H13) in *Arabidopsis* resulted in transgenic plants possessing short root hairs [159,160,161]. Overexpression of the P4Hs displayed long root hairs. These Hyp residues will then be glycosylated depending on the subgroup of HRGPs. The Hyp residues in the EXTs backbones are mainly O-arabinosylated with the exception of a probable single Gal residue [292]. Mutants of the different arabinosyltransferases, responsible for the addition of Ara to EXTs, resulted in short root hairs. Mutations in O-glycosylation processing genes of extensins mainly displayed short root hairs as was seen for the *p4h* mutants [159,160]. In *Arabidopsis*, the transfer of the first arabinose residue to Hyp is performed by at least three hydroxyproline O-arabinosyltransferases (HPAT). The single mutants *hpat1*, *hpat2* and *hpat3* displayed a short-root-hair phenotype [161]. The transfer of Ara molecules to the Ara on Hyp is mediated by several genes: *reduced residual arabinose1-3* (*rra1*, *rra2* and *rra3*), *xylo-endoglucanase 113* (*xeg113*) and *extensin arabinose deficient transferase* (*ExAD*) [164]. Mutation of the genes also resulted in reduced root hair growth [95]. The serine O-α-galactosyltransferase (SGT) catalyses the transfer of galactose to serine residues in extensins. The *sgt1* mutant had longer roots. It was suggested that the phenotype is the result of improved cell extension [91].

Similar to the mutations of O-glycan modifying proteins of EXTs, mutants lacking some enzymes that process AGP O-glycosylation displayed developmental and morphological deviations in the roots [49]. Phenotypical analysis of AGP specific GALT mutants exhibited defective root hairs. Root hair growth was reduced and the root hairs were less dense compared to wild type plants. In some mutants, the *galt* mutants exhibited enhanced salt sensitivity. There was a reduction in root elongation and swelling of the root tip occurred [167,168]. However, other Hyp-O-GALT genes (HPGT) displayed different morphological alterations. The double mutant *hpgt2 hpgt3* and triple mutant *hpgt1 hpgt2 hpgt3* produced longer lateral roots. Additionally, the double and triple mutant developed longer and more densely packed root hairs [166]. Another example showcasing the impaired root growth due to under-glycosylation of AGPs is REDUCED ARABINOSE YARIV1 (RAY1). The primary root of the *ray1* mutant was shortened [165].

#### 3.1.3. Biotic Interactions at the Root Require Some Sweet Regulation

Protein glycosylation in the root is vital to allow the plant to interact with its surroundings. The colonization of the root system by beneficial micro-organisms improves the supply of water and nutrients to the plant [293]. Symbiotic structures such as nodules and arbuscules will be formed to ensure the exchange of nutrients between the two symbionts and to house the beneficial micro-organisms [294,295]. Despite the fact that both partners benefit from the relationship, the development and maintenance of the symbiotic interaction is labor intensive. In case of nodule formation, the number of nodules is strictly regulated through the use of the autoregulation of nodulation pathway [296]. This pathway enables the production of specific glycosylated peptide hormones, including Rhizobia-Induced CLAVATA-Endosperm Surrounding Region (ESR)-related (CLE) 1 and 2 that are found in different species such as soybean (*Glycine max* L.), pea, common bean (*Phaseolus vulgaris* L.) and barrel clover (*Medicago truncatula* L.) [297,298,299]. These CLE peptides are O-glycosylated and require the presence of O-glycans to inhibit nodulation. The functional role of tri-arabinosylation of CLE peptides for autoregulation of symbiosis was confirmed in barrel clover and common bean [299,300]. However, some of the CLE-peptides do not require the arabinosylation [300,301]. Similar feedback systems are found in the symbiosis of arbuscular mycorrhizae in tomato plants that require the Hyp O-arabinosyltransferase enzyme FASCIATED INFLORESCENCE to suppress mycorrhizal colonization [278].

Plant roots encounter not only beneficial bacteria and fungi, but also soilborne pathogens. This exposure results in the development of defense mechanisms. Cell wall proteoglycans belonging to the HRGP family, are enriched in the roots of different species [82,302]. These proteoglycans are involved in the microbial interaction with the root system [303,304]. Recently, it was shown that EXTs that are part of the HRGP family are involved in the defense mechanism of roots by limiting pathogenic oomycete invasion through remodeling the cell wall. Moreover, it was suggested that the arabinosylation of EXTs is vital for strengthening the cell wall during the immune response [82].

Carbohydrate–protein interactions are important in biotic responses of the plant. In case of plant–pathogen interactions, the glycosylation can either be advantageous or detrimental for the host and present on the micro-organism or the host plant [305]. The protein–carbohydrate interactions occur through specific plant receptors, such as receptor-like kinases and receptor-like proteins, and host lectins are of importance to trigger several signaling cascades, e.g., pathogen/microbe-associated molecular pattern triggered immunity or even direct inhibition of pathogen growth [43,305,306,307]. The importance of carbohydrate structures in plant–pathogen interactions are beyond the scope of this review [26,305,308].

### 3.2. Phenotypical Disturbances in Leaves

#### 3.2.1. Abnormal O-Glycosylation: Leaves in Distress

Aberrant glycosylation affects several stages of plant development, resulting in a clear phenotype of the transgenic plant. Although the majority of these phenotypes have been reported in plants with aberrant O-glycosylation, there are also some examples of leaf phenotypes in plants with abnormal N-glycosylation. For instance, the morphological analysis of transgenic plants with a mutation in the ALG10 gene, displayed inhibited growth and smaller leaves [118]. Dwarfism is observed regularly in transgenic plants with N-glycosylation defects [48,125].

Mutants displaying abnormal O-glycosylation, exhibit some peculiar phenotypes. Silencing of several P4Hs (P4H1, P4H7 and P4H9) in tomato resulted in an increase in leaf surface area. The larger tomato leaves obtained through the virus induced gene-silencing, were due to either enhanced leaf epidermal cell expansion or cell division [158].

In *Arabidopsis*, the loss-of-function mutant *sgt1* displayed a larger rosette compared to wild type plants. The reason that the mutant exhibited this leaf phenotype was probably through enhanced cell extension [91]. EXT O-glycosylation also involves different arabinosyltransferases to add 4–5 consecutive arabinose units to the Hyp residues [292]. Loss-of-function of the individual *HPAT* genes encoding these enzymes did not result in any obvious leaf phenotype, suggesting protein redundancy. Impairing the expression of both *HPAT1* and *HPAT2* resulted in a decreased number of leaves. Moreover, the chlorophyll content in leaves was also reduced, inferring early senescence of the leaves [162]. The addition of the third Ara molecule to the Ara chain in EXTs is catalysed by the enzyme named XEG113 [49,95,292]. The mutant *xeg113* exhibited an altered leaf morphology, showing larger petioles [163]. The XEG113 homolog, RAY1, also displayed a leaf phenotype after mutation. The *ray1* mutant had a reduced rosette size [165].

Hyp-O-GALT is responsible for the transfer of a Gal residue onto Hyp residues of AGPs. Eight Hyp-O-GALTs (i.e., GALT2-6 and HPGT1-3) in *Arabidopsis* have been characterized and catalyse the transfer of Gal onto Hyp residues [169]. The single mutants did not display any aberrant leaf phenotype, with the exception of the *galt6* mutation that resulted in accelerated leaf senescence in *Arabidopsis*. The leaves displayed premature yellowing, and chlorosis was due to the reduction in chlorophyll and protein content [167,168]. The phenotype of *galt6* resembles the altered phenotype observed in *hpat1*/*hpat2* [162]. The quintuple mutant *galt2*/*galt3*/*galt4*/*galt5*/*galt6* had fewer and smaller rosette leaves [169]. The triple mutant *hpgt1*/*hpgt2*/*hpgt3* displayed a dwarf phenotype with smaller rosettes which is in line with the phenotype observed for the quintuple mutant [166]. The double mutant *galt2*/*galt5* exhibited a larger number of leaves [168]. It remains unclear why there is such a difference in phenotype between the double and the triple/quintuple mutants.

The enzymatic process of transferring a single β-N-acetylglucosamine/fucose onto hydroxyl groups of proteins can occur in the cytoplasm, the nucleus and the mitochondria. Several roles have been inferred to be related with O-GlcNAcylation and O-fucosylation [86,309]. In general, impairment of genes responsible for the transfer of O-GlcNAc and O-Fuc on proteins affected diverse plant processes [90]. Moreover, proteomic analysis using *Arabidopsis* demonstrated the relevance of O-GlcNAc modification. Approximately 262 proteins were shown to carry the monosaccharide decoration. Several of these modified glycoproteins play a role in cellular regulation, such as signal transduction and gene regulation [86]. In *Arabidopsis*, O-GlcNAcylation is performed by SEC. Mutations in the SEC gene caused a reduction in the number of leaves at flowering, as described earlier. Generally, the *sec* plants produced leaves at a reduced rate [258]. SPY was previously regarded as a O-GlcNAc transferase but it was recently demonstrated that SPY is an OFT [152]. O-fucosylation also affects leaf morphology such as the reduction in leaf serration size and decreased number of leaf serrations. Moreover, leaf growth was also impaired [151,154,255]. Aberrant phenotypes due to the mutation of SPY in *Arabidopsis* can be attributed to alterations in plant hormone responses. Indeed, SPY interacts in several responses of cytokinins and GA, but it is more complicated due to the fact that SPY also has GA-independent roles in the plant [90].

The premature senescence leaf (PSL) locus from rice encodes a putative β-1,6-GlcNAc-transferase and is suggested to be involved in protein glycosylation. Deletion of a phenylalanine residue in PSL gave rise to *psl* mutants, characterized by early leaf senescence and enhanced production of ethylene. Senescence was caused by accumulation of reactive oxygen species (ROS) and an apoptotic response. Furthermore, *psl* mutants showed downregulated O-GlcNAcylation but increased transcription of S-adenosyl methionine synthetase, required for ethylene biosynthesis. The obtained results suggest a relationship between O-GlcNAcylation, leaf senescence and ethylene signaling [155].

Some specific glycoproteins are also involved in leaf development. For example, the inactivation of the glycoprotein AGP19 displayed a dwarf phenotype in *Arabidopsis*. The leaves of these transgenic plants *agp19* were round and smaller. Additionally, the rosette leaves contained reduced levels of chlorophyll and anthocyanins [310].

#### 3.2.2. Specialized Leaf Tissues Are Annoyed by Absent Glycosylation

Trichomes are protuberances on the aerial part of the plant that differentiated from epidermal cells [311] and play different roles in plants including plant defence [311,312]. Trichomes have been linked to both abiotic and biotic stress responses. Water stress, temperature variations (i.e., both heat and cold), photosynthesis regulation and detoxification of the plant through biomineralization and secretion have been implicated to be modulated by trichomes [312,313,314]. Trichomes are mainly associated with biotic stresses and prevention of herbivore attacks because they contain insect and herbivore deterrents and/or toxins, allowing insect reduction and immobilization [311,312,315]. Molecular genetic analysis using mutants revealed that both trichomes and root hairs develop through similar molecular mechanisms. In the chapter concerning roots it was already shown that aberrant glycosylation influences the development of the root hairs [316]. Similarly, aberrant protein glycosylation can affect the architecture and development of trichomes.

Phenotypical analysis of a transgenic *Arabidopsis* plant overexpressing the *P4H1* protein responsible for the initiation of O-glycosylation of proline, did not show any major aberrant phenotype. Despite the normal growth, the plants did not develop any trichomes [157]. Functional characterization of three β-glucuronosyltransferases (GLCATs) through mutational analysis showed that trichome branching and size were reduced. GLCATs mediate the transfer of glucuronic acid (GlcA) to AGPs. More specifically, the double mutant *glcat14a*/*glcat14b* and triple mutant *glcat14a*/*glcat14b*/*glcat14c* displayed reduced trichome size and branching [317]. The abnormal trichome phenotype was attributed to the reduced Ca^2+^ binding of GlcA in the glycan moieties of AGPs. The reduced GlcA levels decreased the Ca^2+^ levels necessary to trigger growth responses in these tissues [317,318,319]. Additionally, the nuclear-localized OFT SPY also interacts with trichome development. Mutation in the SPY gene caused enhanced branching of trichomes [153]. The enhancement of branching was attributed to the alterations in plant hormone homeostasis and signaling in *spy* plants [90].

## 4. Functionality of Protein Glycosylation

As discussed above, impaired protein glycosylation clearly affects plant development and phenotype. The question arises as to what is the true contribution of various types of glycosylation for the functionality of glycosylated molecules. While high mannose N-glycans are important for protein folding, intracellular trafficking and quality control in the ER, much less is known with respect to the function of more complex N-glycans. O-glycosylation mainly defines the function of some cell wall related proteins [7,67]. At present, there are only a few specific glycosylation events that can be linked to a particular protein function.

DELLA proteins cause growth repression through transcriptional reprogramming of different genes involved in cell division, expansion and differentiation. The change in transcription levels for these genes is modulated through protein–protein interactions between DELLA proteins and several transcription factors [320,321,322,323]. REPRESSOR OF GA1-3 (RGA), a DELLA protein, is SPY-dependent O-fucosylated. It was reported that RGA extracted from the *spy* mutant displayed reduced binding activity compared to the DELLA protein obtained from wild type plants [152]. Additionally, it was shown that O-GlcNAcylation by SEC inhibited the interaction of RGA with four of its interactors, suggesting opposite roles for O-GlcNAcylation and O-fucosylation in RGA [152,320]. These results provided evidence that O-fucosylation and O-GlcNAcylation influence specific functions of RGA.

Another example showcasing the importance of glycosylation for a particular protein, is the endo-β1,4-glucanase glycoprotein KORRIGAN1 (KOR1), necessary for cellulose biosynthesis. Recombinant KOR1 with mutated N-glycosylation sites yielded proteins with reduced glucanase activity [324]. In addition, aberrant N-glycosylation of KOR1 also changed its localization in the different subcellular compartments [289,290].

Despite the considerable number of studies focusing on elucidating the biological role of glycosylation enzymes, their characteristics and respective phenotypes, less attention is paid in performing analyses for specific glycoproteins. Only a few examples exist where glycosylation functionality is approached at the level of a single glycoprotein. Two striking examples include the MTR1 glycoprotein and the SCR/SRK glycoprotein, which are important for flower development and reproduction [173,183]. Site-directed mutagenesis of the N-glycosylation sequon in MTR1 revealed that this modification is crucial for normal anther development and pollen fertility. Its role was confirmed with subsequent analysis of the glycosylation profile through PNGaseF treatment [173]. Similarly, the extent of glycosylation of the SCR/SRK tandem determined the pollen haplotype, allowing the stigma to discriminate between self and non-self and henceforth evoking self-incompatibility [183]. Similarly, glycosylation of pattern recognition receptors is required to mediate plant immune signaling [325].

All examples above illustrate how protein glycosylation can really make a difference for the plant. At present, the large majority of papers that study the importance of glycosylation make use of overexpression lines or mutant lines for enzymes involved in the glycosylation process. Since these mutations affect the glycosylation profile of a multitude of proteins, these studies are often difficult to interpret and do not allow us to draw conclusions with respect to the importance of glycosylation for one particular protein. Indeed, knock-down and knock-out lines of certain glycosylation enzymes yield plants that show combined effects of aberrant glycosylation in a diverse set of proteins. Therefore, a better way to address functionality of glycosylation is by site-directed mutagenesis of the amino acids that are part of the glycosylation sequon [324]. In case the protein of interest exhibits a specific biological activity, the protein can be extracted and/or purified to perform in vitro activity assays [152,320]. Deglycosylation enzymes and chemical inhibitors of glycosylation such as tunicamycin [326], can also be useful to obtain deglycosylated proteins. At present different technological challenges for the study of plant glycosylation still exist, such as mapping and characterization of N- and O-glycans. Biochemical studies can be complemented with in planta studies, such as genetic screens, mutant collections to study protein functions. Recently, fluorescent timer proteins have been introduced as a nice tool to study protein dynamics and these can also be used in the study of plant glycobiology [67,286]. All these advanced techniques can ultimately help to decode the biological function of protein N- and O-glycosylation in plants.

## 5. Glycolipids and Plant Development

### 5.1. Roles of Glycoglycerolipids and Lipid Profile Alterations

The class of glycoglycerolipids consists of different lipids such as galactolipids, glucuronosyldiacylglycerol and sulfoquinovosyldiacylglycerol [102]. The galactolipids, mainly monogalactosyldiacylglycerol (MGDG) and digalactosyldiacylglycerol (DGDG), are the predominant glycerolipids in chloroplast membranes and constitute the majority of the total membrane lipid [103,327,328]. The galactolipid profile differs between plants and tissues [329]. The composition and galactolipid content in the membrane influences membrane stability and functional activity of membrane proteins [103]. In favorable growth conditions, galactolipids are retained in the plastid membranes, but their function is not exclusively related to the chloroplasts [328]. MGDG and DGDG are associated with the photosynthetic apparatus but are also of importance within non-photosynthetic organs such as the roots [103,328]. The ratio of MGDG and DGDG is implicated in the stable maintenance of the thylakoid membranes and sustain photosynthesis [328]. A stable DGDG/MGDG ratio in leaf tissues indicates favorable growth conditions [329,330,331]. Besides the galactolipids, glucuronosyldiacylglycerol (GlcADG) and sulfoquinovosyldiacylglycerol (SQDG) also occur in the thylakoids of the chloroplast [327]. The structure and synthesis of the glycoglycerolipids has already been reviewed [327,330].

In the chloroplast, the function of glycoglycerolipids, together with other lipids (e.g., phosphatidylglycerol), consists of compartmentalization and maintenance of the dynamic network that embeds the protein complexes. Next to providing the matrix for the photosynthetic protein complexes, both MGDG and DGDG are an important part of the protein complexes [103,327,330,332,333]. More specifically, MGDG is present in photosystem (PS) I, photosystem II, and the Cytochrome b6f complex, and DGDG is present in PSII and the Light Harvesting Complex II [332]. A knock-down mutation of the MGDG synthase 1 in *Arabidopsis*, one of three MGDG-synthesizing genes, resulted in impaired MGDG synthesis [334,335,336] and influenced the structural organization of the chloroplast of light-grown plants. The mutated plants had a chlorotic phenotype [335]. The lipid profile in chloroplasts typically changes due to different environmental cues such as drought, freezing and nutrient deprivation [327,330]. Furthermore, the Brittle stem and Zebra leaf (BZ1) locus from rice, encoding a putative UDP-Gal/Glu epimerase was recently reported to be associated with mechanical strength and coloring of leaves. Mutant *bz1* plants were characterized by phenotypes with abnormal cell walls and chloroplast membranes. On a proteomics level, mutant plants showed reduced AGP O-glycosylation as well as a significantly reduced amount of MGDGs. On the other hand, rice plants overexpressing BZ1 showed significant growth enhancement. The obtained results point towards a role of BZ1 in the synthesis of MGDGs and AGPs through UDP-Gal supply in the cell wall and chloroplast membranes [156].

Studies observing the effect of drought on the galactolipid content in leaf tissues revealed differences between species and cultivars [102,329,332,337,338,339]. The total galactolipid content decreased during water deficit in both tolerant and susceptible wheat (*Triticum aestivum* L.) and barley (*Hordeum vulgare* L.) plants but during rehydration the recovery of tolerant plants was much faster [337]. Similarly, in a susceptible cowpea (*Vigna unguiculata* L.) cultivar the total galactolipid content dropped during water deficit, whereas the galactolipid content remained relatively stable within the tolerant cultivar [338].

The acclimation of plants towards heat stress is also correlated with changes in lipid composition. In response to temperature stress, plants alter their membrane characteristics to optimize the cellular processes such as photosynthesis [340]. Elevated temperatures result in an increase in saturation of leaf fatty acids, polygalactosylation of galactolipids, as well as increase in triacylglycerols [341,342]. Additionally, the decrease in the ratio of MGDG/DGDG was shown to be of importance to improve thermotolerance in *Arabidopsis*, common bean and wheat [340,343,344]. However, no significant changes of DGDG and MGDG were observed in *Arabidopsis* plants [342,345]. Analysis of *Arabidopsis* mutants in the DGDG synthase 1 gene (DGD1) showed that basal thermotolerance was impaired. Despite heat acclimation, the mutants were still prone to heat stress [340]. In wheat leaves, the SQDG content decreased at high temperatures in both tolerant and susceptible cultivars [344].

Similar to heat stress, freezing temperatures also contribute to the polygalactosylation of galactolipids [346,347]. The synthesis of oligogalactolipids is mediated by SENSITIVE TO FREEZING 2 (SFR2) in *Arabidopsis*. This gene is a galactolipid:galactolipid galactosyltransferase (GGGT) and removes a Gal from MGDG to decorate another galactolipid such as MGDG. The galactosylation is performed in a processive manner, resulting in the formation of oligogalactolipids. In addition, diacylglycerol (DAG) is produced and used to synthesize triacylglycerol (TAG) [327,346,348]. *Sfr2* mutants show no phenotypes under normal growth conditions. Applying freezing stress to *sfr2* mutants causes severe damage to the leaves [349,350].

The depletion of nutrients such as nitrogen and phosphate had severe consequences for membrane lipid composition. In the case of phosphate starvation, an increase in DGDG, GlcADG and SQDG were observed [327,351]. The rise of the phosphorus-free glycoglyercolipids during phosphate deprivation is attributed to the substitution of phospholipids with DGDG, GlcADG and SQDG [327,351]. The conversion of phospholipids occurs in two steps: (1) hydrolysis of phospholipids that yield phosphate and diacylglycerol (DAG) and (2) glycoglycerolipid biosynthesis [351]. The replacement is not only restricted to the plastid membranes but also to extraplastidial membranes. The presence of DGDG was observed in the plasma membrane of oat (*Avena sativa* L.) and common bean and in the mitochondria and tonoplasts of *Arabidopsis* [351]. Next to phosphate deprivation, the absence of nutrients such as nitrogen and magnesium in *Arabidopsis* also influenced the lipid profile. When plants are grown in the absence of nitrogen, the abundance of phospholipids and DGDG was increased but MGDG decreased. Nitrogen and magnesium starvation resulted in the reduction of the MGDG/DGDG ratio [352].

### 5.2. Glycosylated Protein-Lipid Anchors: Glycophosphatidylinositols

Glycophosphatidylinositols (GPIs) are glycolipids ubiquitously present in eukaryotes ranging from protozoa and fungi to humans and plants [353,354,355]. The class of GPIs is a heterogeneous cluster of molecules. This diversity of GPIs arises from the wide variety of lipids and substitutions that can occur on the conserved core structure. This conserved GPI core is found throughout different eukaryotic lineages [356,357] and is composed of an inositol phospholipid, one non-N-acetylated glucosamine, three Man and a terminal ethanolamine phosphate [354,356]. The lipid moiety can consist of either a glycerolipid or a sphingolipid. Despite the elucidation of the GPI structure throughout the different kingdoms, only one GPI molecule has been fully resolved in plants [356,357]. The GPI structure reported in pear suspension cells resembles the core structure from other eukaryotes with the exception that it has a β-galactosyl substituent [358]. The core glycan structure has also been confirmed in *Arabidopsis* [357].

The biosynthesis of the GPI core and its substitutions in mammals and in yeast has been studied extensively, but knowledge on the GPI core assembly in plants is still limited [356,357,359]. Nevertheless, insight into the GPI glycan core assembly has recently been provided by Beihammer et al. [357]. Homologs of genes involved in the biosynthetic steps of GPI core assembly have been found in *Arabidopsis*. Mutants of these homologs such as APTG1, SETH1, SETH2 and PEANUT1 display defective phenotypes. The importance of GPIs is evident through the embryo lethality and reproductive defects that occur in plants, which are unable to synthesize the structure [120,239,355,360,361].

Compared to other glycolipids, GPIs are mainly attached to proteins [353,354,355]. To date, no protein-free GPIs have been reported in plants. The abundance of GPI-anchored proteins (GPI-AP) within 29 eukaryotic proteomes was predicted to range between 0.21% and 2.01%. More specifically, the estimated amount of GPI-AP in *Arabidopsis* is 0.83% [362]. The predicted abundance of GPI-AP is probably an underestimation due to the process of alternative splicing [355,363,364]. The GPI structure is assembled at the ER membrane. Afterwards, the GPI anchor is covalently bound to the carboxy termini of proteins and targets proteins to the cell surface [356,365]. The post-translational addition of GPI to proteins, known as protein glypiation, anchors these proteins to the outer leaflet of the plasma membrane [354,356]. GPI-AP are regularly located in membrane microdomains, enriched in sphingolipids and sterols [356]. The predicted GPI-APs have been reported both in the plasma membrane and in the membrane microdomains [366].

Proteins associated with the GPI-anchors have very diverse functions. Cell wall synthesis/maintenance proteins, proteases, receptor-like proteins and lipid transfer proteins are some examples of the rich library of proteins that can be glypiated [301,355,367]. These GPI-APs manage crucial processes in the plant, including plant development, reproduction, cell wall metabolism, cell polar expansion, stress responses, hormone signaling, pathogen responses [356,359]. AGPs, COBRA proteins, LORELEI proteins and some receptor-like proteins, that are modified with a GPI, have been studied in more detail. The LORELEI proteins interact with RLKs to participate in ligand recognition. Additionally, LORELEI is responsible for chaperoning the RLK towards the plasma membrane [355].

### 5.3. Glycosphingolipids: Sugar Coating the ‘Sphinx’

Sphingolipids are omnipresent in eukaryotes and even occur in some bacteria [368]. Despite their widespread occurrence in eukaryotes, the molecular moieties of the sphingolipids differ between kingdoms [369,370]. For example, glycosyl inositol phosphoryl ceramides (GIPCs) are restricted to plants, fungi and some protozoans [371], while the glucosylceramides (GlcCERs) are common in all eukaryotes [369,372]. In general, sphingolipids consist of a long chain amino-alcohol base (LCB), also known as the sphingoid backbone [373]. The backbone is generally composed of 18 carbon atoms [374]. The amino group of the LCB is acylated with a fatty acid to form a ceramide. This structure forms the cornerstone for more complex sphingolipids, in which the ceramide can undergo further modifications of both the LCB and the fatty acid. Aside changes to the lipid moiety, conjugation of the hydroxyl group of the sphingoid backbone through glycosylation, phosphorylation or addition of an inositol-phosphate group (IPC), increases the complexity and diversity of sphingolipids [374,375]. The different modifications confer the potential of sphingolipids to act both as integral structural components in the membranes and as bio-active moieties involved in cellular processes [376].

Generally, plant sphingolipids are classified into four major groups: ceramides, GlcCERs, GIPCs and free long chain bases [373,375]. In the last few decades, the plant sphingolipids were studied in more detail [370,372,377]. These molecules occur in the plasma membrane, tonoplast and other endomembranes [378]. The abundance of sphingolipids is higher in the outer leaflet of the plasma membrane and thus contributes to the lipid asymmetry of the plasma membrane [372,379]. The plasma membrane of tobacco cell suspension cultures (Bright Yellow 2) contains 30–40 mol% GIPCs of the total lipids [369]. Moreover, sphingolipids together with sterols are enriched in specific domains in the plasma membrane called lipid rafts but are also increased in the plasmodesmata. Next to the lipids, lipid rafts and the plasmodesmata contain a multitude of proteins either transmembrane proteins or GPI-anchored proteins [369,380,381,382]. The association of these lipid domains is influenced by the biosynthesis of sterols and sphingolipids. Sterol and sphingolipid biosynthesis affect raft organization and the function of proteins occurring in the assembly. For example, the disruption of sterol and sphingolipid homeostasis, resulted in aberrant localization of GPI-APs involved in callose turnover [383].

To date, the main glycosphingolipids found within plants are GlcCERs and GIPCs [372,384]. In *Arabidopsis* leaves, both glycosphingolipids, GIPCs and GlcCERs, represent approximately 98% of the total sphingolipid content [384]. The percentage displays the sheer importance of these molecules. The polar head structure of GIPCs, consists of a hexonic acid (HexA) and a hexose glued together by a hydroxyl, amine or a N-acetyl group. HexA is linked with the phosphatidyl group. The polar head of GIPCs can be composed of diverse saccharides such as Gal, Glc, Ara, Man and Fuc [380]. The carbohydrate chain of GIPCs in 23 species, including algae, mosses, ferns, *Gymnospermae* and *Angiospermae*, was characterized and revealed that the glycan moieties of the sphingolipids differ between plant species and tissues [371,385]. An interesting example is the knock-out mutant of GLUCOSAMINE INOSITOLPHOSPHORYLCERAMIDE TRANSFERASE1 (GINT1) in rice and *Arabidopsis*. GINT1 is responsible for the glycosylation of a subgroup of GIPC. Its mutation was lethal in rice while the mutation of *GINT1* did not affect vegetative growth of *Arabidopsis* [386]. In plants, the additional glycosylation of GlcCER can occur. Di- and triglycosylceramides have been identified in plant lipid extracts but these represent a minor fraction [387].

Biosynthesis of sphingolipids is conserved within eukaryotes [375,377]. The glycosylation of ceramides and the transfer of phosphatidylinositol on ceramides occur in the ER and in Golgi, respectively. Disruption of glycosylation of GIPCs can cause growth defects or even lethality [371]. Knock-out mutants of the genes responsible for the glycosylation of sphingolipids such as GlcCER synthase and inositol phosphorylceramide glucuronosyl transferase 1 are lethal or result in changed phenotypes. These knock-out mutants highlight the importance of the glycosylation of sphingolipids [370]. The processes involved in the biosynthesis of plant sphingolipids are beyond the scope of this review. For more detail, we refer to some recent publications [373,374,388].

Glycosphingolipids serve as a hydrophobic barrier between the environment and the cell, similar to other plasma membrane lipids. Moreover, these lipids also contribute towards a multitude of other functions. There is functional evidence for the involvement of GlcCERs in (post-Golgi) protein trafficking and Golgi morphology, resulting from studies using mutants and inhibitors of the GlcCER synthase. Treatment with the GlcCer biosynthesis inhibitor, DL-threo-1-phenyl-2-decanoyl amino-3-morpholino-propanol (PDMP), resulted in the disintegration of Golgi stacks into vesicular structures and in the disturbed transport of membrane proteins [389,390]. Glucosylceramides have been implicated in both chilling/freezing tolerance and in drought tolerance [375,378]. During cold acclimation experiments in both rye and oat, the glycosphingolipid composition of the plasma membrane was altered, with reduced levels for GlcCER [391]. Similar responses to cold stress were also recorded in other plants [372]. Hypoxia stress induced enhanced levels of several sphingolipids including GIPCs and GlcCERs [392,393]. These GIPCs have also been implicated in the wall-membrane attachment. The interaction between membrane and cell wall should occur through a boron bridge linking GIPC and rhamnogalacturonan II [394]. GIPCs are also linked to high salinity stress response. Ca^2+^ levels in the cytosol increase and subsequently trigger the SOS pathway [395]. Mutation in the allele of IPUT1, *monocation-induced [Ca^2+^] increases 1* (*moca1*), was unable to increase cytosolic Ca^2+^-levels [396]. Moreover, GIPCs can be hijacked by plant pathogens to facilitate infection. Bacterial, fungal and oomycete plant pathogens produce cytolytic toxins that interact with a subgroup of GIPCs present in eudicots, but not in monocots [397].

## 6. Sugar Signaling as the Puppet Master of Plant Development

Plants as autotrophic organisms produce sugars in mature photosynthetic leaves (source organs) which are subsequently used to support the storage and growth in sink tissues such as the roots, young leaves and fruits. Apart from driving growth as metabolic substrates, sugars also serve as signaling entities, interacting with environmental stimuli and metabolic signals to regulate cell growth through the remobilization of nutrients and storage compounds [398,399]. Free soluble sugars like Glc and Fru are thus directly related to the cellular energy status of the cell. As such, sugars have been linked to the regulation of growth and stress responses, but also as important cues during developmental decisions such as germination, vegetative phase transition, flowering and senescence [400,401,402,403]. However, the interplay between soluble sugars and glycoconjugates in plants remains largely underexplored.

### 6.1. Sugar Signaling through the SnRK1/TOR Nexus

**Figure 6:**
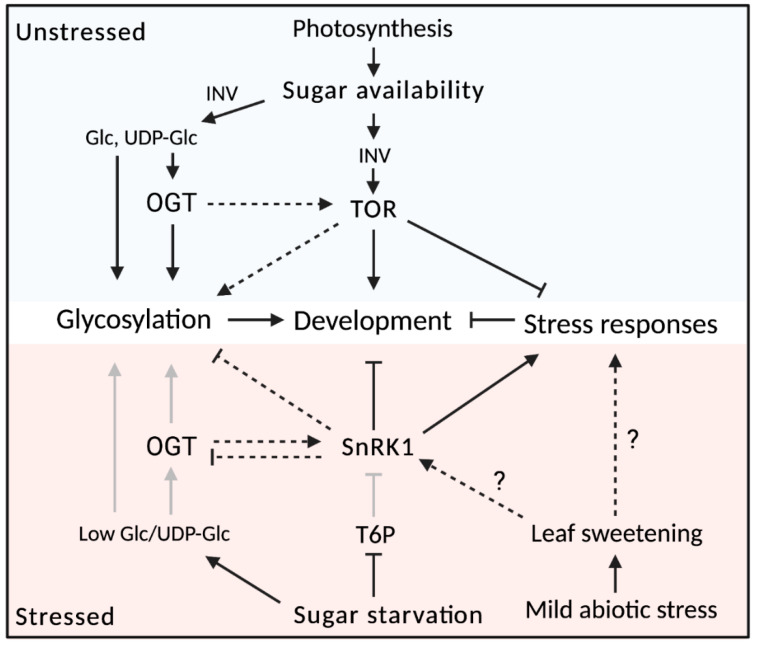
Scheme illustrating the links between sugar metabolism/signaling and glycosylation and the trade-off between growth and stress responses. The figure illustrates the effect of sugar metabolism and signaling during unstressed conditions and stressed conditions in terms of sugar availability. Under non-stressed conditions, photosynthesis provides sugars to be used in metabolism and directly in glycosylation reactions. Sugar availability stimulates Target of Rapamycin (TOR), promoting growth and development and inhibiting stress responses. Sugar availability can also directly influence glycosylation by serving as the substrates, for instance through O-GlcNAc transferases (OGTs). Invertases (INVs) are key components, specifically in sink tissue, to convert sucrose to metabolizable hexoses. Under these conditions, Snf1-related protein kinase 1 (SnRK1) is inhibited by trehalose-6-phosphate (T6P). When exposed to severe stresses, photosynthesis is hampered, resulting in sugar deprivation, reflected through T6P levels (mirror sucrose levels). Low T6P results in an active SnRK1, inhibiting growth and possibly also glycosylation by regulating OGTs as is the case in animals. Active SnRK1 also promotes stress responses. It is important to note that mild abiotic stresses result in temporal leaf sweetening which can also potentially stimulate SnRK1 and/or stress responses. During sugar deprivation, TOR is typically inactive. Apart from sugar signaling pathways, limited sugar availability can also directly hamper glycosylation due to limited substrate availability and low energy levels. It is unclear whether a sugar sensor exists that translates sugar availability to glycosylation reactions in plants; however, candidates include OGTs themselves or SnRK1, since this appears to be the case in animals. In animals, AMP-activate protein kinase (AMPK), the homolog of SnRK1, and OGTs co-regulate one another to alter their activities and localization. It is important to note that glycosylation will never be completely inhibited even under severe stresses, as a low level of glycosylation is also important during these conditions. Arrows with arrow heads indicate stimulatory effects whereas arrows with blunt ends indicate inhibitory effects. Solid lines are known effects and dashed lines predicted effects. Grey arrows indicate an inactive pathway under a specific condition. This figure was created with BioRender.com.

There are two evolutionary conserved protein kinases in the center of sugar sensing and signaling pathways in plants which regulate energy homeostasis, namely the Snf1-related protein kinase 1 (SnRK1, Snf1 in yeast and AMP-activate protein kinase (AMPK) in animals), and the Target of Rapamycin (TOR) kinase. The general consensus is that these two kinases act in concert, and antagonistically to finetune the trade-off between growth and stress responses [398] (Figure 6). SnRK1 is typically activated by low energy conditions (during stress) and promotes the activation of catabolic processes and inhibits energy-demanding anabolic processes and growth. During stress, the SnRK1 kinase is inhibited by sugars [404,405], in particular by the sugar phosphates trehalose-6-phosphate (T6P) (reflecting the cellular Suc status), glucose-1-phosphate (G1P) and glucose-6-phosphate (G6P) [406,407,408]. In contrast to SnRK1, TOR promotes growth and biosynthetic processes when energy is sufficient [409], like high Glc levels [410]. The mechanisms by which sugar availability regulates SnRK1 and TOR pathways remain a topic of debate. The downstream responses of these two kinases are generally associated with large-scale reprogramming of transcriptional networks and metabolic readjustments. With these two kinases acting as main regulators of plant metabolism, it is no surprise that they are also vital components in the regulation of developmental processes of plants.

### 6.2. Developmental Regulation by Sugar Signaling

The SnRK1/TOR nexus is an important signaling component that affects plant growth from a very early stage of development. This is evident in pea plants where SnRK1α is silenced at the post embryonic stage, resulting in defective cotyledon development and eventual seed maturation [411], associated with reduced accumulation of protein reserves, impaired desiccation tolerance and advanced germination [412]. These developmental defects were attributed to altered expression of genes associated with cell proliferation and differentiation and seed maturation. In addition, these SnRK1 defective plants showed disturbances in cytokinin and ABA levels, pointing to interplay between SnRK1 and ABA and auxin/cytokinin pathways [411]. Similarly, *Arabidopsis* plants over-expressing SnRK1α1 showed delayed seed germination [413].

A recent study showed that SnRK2, a main driver of ABA-triggered stress responses in fact has a dual function in regulating SnRK1 and growth [414]. Under optimal conditions, SnRK2 promotes growth by interacting with SnRK1, preventing SnRK1 to inhibit TOR. During stress (presence of ABA), the SnRK2 and SnRK1 complex dissociates, releasing SnRK1 to activate stress responses and inhibit growth. It was further shown that SnRK1 positively regulates embryogenesis, seed yield and growth during high temperatures in *Arabidopsis* [415]. The cellular sugar status reflected through T6P is likely an important component during these processes, since it was recently shown that T6P promotes seed filling by activating auxin biosynthesis in pea plants [416]. In contrast, TOR knock-out plants show early embryo arrest, and impaired cell division leading to abolished endosperm development [417]. These abnormalities are supported by the expression of TOR in developing seed tissues and emphasize the importance of this kinase in cell division and cellularization. This is further supported by findings indicating that a mutation in the REGULATORY-ASSOCIATED PROTEIN OF TOR (RAPTOR) gene leads to embryo lethality. TOR is further believed to regulate growth at later stages of development, promoting seed size and longevity [418,419].

Early seedling development through cell elongation heavily relies on seed reserves, followed by the initiation of meristem activity and cell division. This phase transition is most probably linked to the availability of photo-assimilates, seeing that sugar feeding is able to support growth and organ formation [420]. TOR activity is required for post-germination growth, since plants lacking a functional TOR complex show reduced growth [419], attributed to the positive effect of TOR on cell division, cell elongation, translation and photosynthesis [421]. Sugar- and light-induced seedling growth is also abolished when TOR is chemically inhibited, and TOR activates the root meristem after germination in a Glc-dependent manner [422,423]. During unfavorable conditions, however, development is arrested through ABA and can also be mimicked by high sugar treatments [41]. Plants over-accumulating SnRK1 are hypersensitive to Glc and ABA, resulting in defective cotyledon development and leaf formation [413,424]. In contrast, growth is arrested in *Arabidopsis* in which SnRK1α is transiently silenced by virus-induced gene silencing, and cannot be rescued by exogenous sugar treatment [404].

Furthermore, a recent study showed that T6P is an important player during the vegetative phase transition in *Arabidopsis* [402]. *Arabidopsis* plants defective in TREHALOSE PHOSPHATE SYNTHASE1 (TPS1) showed a delay in vegetative phase transition by repressing miR156 expression and accumulated significant amounts of Suc. A model was previously proposed where T6P and SnRK1 are involved in the transition from the juvenile to adult phase by interacting with miR156; however, the exact mechanisms remain unclear [413]. It is thus possible that SnRK1, through T6P is an important component to translate the photo-assimilate status to regulators of the transition phase. This is supported by the fact that low sugar availability prevents the transition to the adult phase through high levels of miR156, and sugar supply represses the expression of *MIR156* genes [425,426]. It is interesting that mutants in the OFT SPY show accelerated developmental transitions, related to the O-glycosylation of transcription factors acting downstream of miR156 [427]. This supports the idea that T6P/SnRK1 signaling might be altered by glycosylation events.

Originally, it was believed that shoot branching is inhibited by auxin transport from the shoot apex to the axillary buds (resulting in apical dominance), and that the depletion of auxin when the shoot tip is removed is responsible for increased shoot branching. However, a later study showed that Suc is transported to the axillary buds when the shoot tips of pea plants were removed, resulting in reduced expression of the branching inhibitor Branched1 gene [428]. Additionally, this was dependent on the presence of source leaves, indicating that source–sink relationships determine shoot branching. It was further shown that over-expression of invertases in *Arabidopsis* severely disturbed branching patterns, suggesting that the ratio of hexoses/Suc is an important regulator of branching [429]. Interestingly, apical dominance is enhanced in plants with reduced T6P, and plants overexpressing T6P metabolizing enzymes (TPS) showed increased branching [430,431], suggesting a role for T6P, through SnRK1, in the regulation of branching. More recently, it was shown that *TPS1* is expressed in the axillary buds, subtending vasculature, and in the leaf and stem vasculature [432]. Expressing a trehalose phosphate phosphatase (*TPP*) to reduce T6P in the axillary buds or vascular tissue strongly delayed bud outgrowth in long days and inhibited branching in short days. Higher T6P in the vascular tissue enhanced branching by inducing the expression of FLOWERING LOCUS T and upregulating Suc transporters. This finding directly links T6P (possibly through SnRK1) with the local regulation of bud outgrowth and systemic regulation of branching through FLOWERING LOCUS T. Additionally, *Arabidopsis* plants with mutated forms of the TOR complex showed increased branching, suggesting that TOR inhibits branching [433,434].

It is also important to point out that Glc-activated TOR restricts the plasmodesmata transport in leaves [435]. Here, TOR was significantly more active in mature leaves synthesizing excess sugar than in young growing leaves, correlating with decreased rates of transport through plasmodesmata in mature leaves. Apart from SnRK1 and TOR, hexokinase 1 (*HXK1*) over-expressing plants showed loss of apical dominance [436]. It was confirmed in rose, pea and Arabidopsis that HXK1 promotes shoot branching [437]. These findings show that sugar signaling events control branching of vegetative shoots and inflorescences.

SnRK1 and TOR are also important regulators of flowering. *Arabidopsis* SnRK1α1 over-expressing plants showed delayed flowering under long days [413,438]. Similarly, T6P also regulates flowering, but it is unclear whether this is through SnRK1 signaling pathways. Interestingly, flowering can also be induced in the dark by exogenous sugar treatment, requiring the TOR kinase [420,423]. This is further supported by the late-flowering phenotypes of LETHAL WITH SEC THIRTEEN 8 (LST8) and *raptor* mutants [433,434], suggesting that TOR promotes flowering under normal conditions.

Finally, SnRK1 and TOR are also highly involved during natural and dark-induced senescence, to remobilize nutrients to younger and developing organs. For instance, SnRK1 was shown to extend leaf longevity during senescence [439]. It appears that T6P is involved in senescence, correlating with sugar accumulation. Senescence is delayed in plants over-expressing a TPP to lower T6P levels [403]. These plants also do not show typical senescence induced by sugar treatments. This is consistent with delayed senescence observed in SnRK1 over-expressing plants [439], since T6P inhibits SnRK1. It is interesting that SnRK1 and TOR affect senescence in a similar manner. Defective TOR results in early leaf senescence, and TOR over-expression delays senescence [419]. However, over-expression of the kinase domain of TOR results in early leaf senescence [440], and this is believed to be through the repression of autophagy pathways [398], crucial in the recycling of cellular components during leaf senescence [441]. This is supported by the fact that both SnRK1 and TOR regulate autophagy in plants in response to the cellular sugar status [442,443,444].

### 6.3. Interplay between Sugar Signaling and Glycoconjugates

Apart from the metabolic and signaling roles of small soluble sugars, not much is known about the interplay between sugar signaling and glycosylation events. However, some emerging data suggest that there are in fact overlapping responses shared by these events, particularly in the context of stress (Figure 6). Since the sugar level is a direct indication of the energy status of plant cells, and sugars directly participate in glycosylation reactions, they are important in protein synthesis and post-translational modifications [445]. It is interesting that a large portion of the HXK1, a Glc sensor and the first enzyme in glycolysis, is associated with the Golgi membrane, and is believed to be directly involved in the synthesis of UDP-Glc required for glycosylation reactions in the Golgi [446,447]. Alternatively, it has been proposed that these non-cytosolic HXKs might be involved in the sensing of hexoses during the induction of systemic acquired resistance and the repression of photosynthetic gene expression, and that this hexose sensing is somehow involved in the secretory pathway [448].

In animals for instance, the energy level (Glc) of cells is linked to the unfolded protein response [449], crucial for proper protein modification and folding during stress. Interestingly, it was shown that the activity of a histone-modifying enzyme (OGT) in animals is directly linked to extracellular Glc concentrations [450]. Although the implication of histone GlcNAcylation remains unclear, it is likely to have significant transcriptional effects. Thus, nutrient sensing by OGT may be important in the modulation of chromatin remodeling and the regulation of gene expression [451]. Glc deprivation in rice ER-LOCALIZED ADENINE NUCLEOTIDE TRANSPORTER 1 (ER-ANT1) mutants unable to transport ATP into the ER showed induced expression of SnRK1 [452], likely to limit energy consuming reactions and protein synthesis due to the inability to properly maintain the secretory pathway. This was supported by the activation of UPR responses in these plants, suggesting that SnRK1 might be activated upon defective protein glycosylation. Evidence for the interplay between SnRK1 and UPR pathways, especially with ethylene and ROS as intermediate factors are starting to emerge [288]. Although most of the findings are in a stress context, it shows that the triad SnRK1/ethylene/ROS is heavily involved to maintain proper glycosylation events during altered growth conditions.

To this point, a direct role for SnRK1 upstream of the secretory pathway acting as energy gauge has not been described. However, it is possible that when plants are exposed to stresses leading to sugar deprivation that SnRK1 might negatively regulate protein glycosylation in an attempt to conserve energy. In animals, the SnRK1 homolog, AMPK, phosphorylates and alters the activity, substrate specificity and cellular localization of OGT in several cell lines [453]. Additionally, it was shown that both the α- and γ-subunits of AMPK are O-GlcNAcylated, increasing AMPK activity [453]. The authors suggested that OGT and AMPK cooperatively regulate nutrient-sensitive intracellular processes that mediate metabolism, growth, proliferation and tissue function. Seeing that these kinases are largely conserved between the different kingdoms of life [405], it is likely that these regulatory processes are conserved in plants (Figure 6).

Besides Glc, UDP-Glc, the direct substrate for glycosylation might also act as an indicator of sugar availability (Figure 6). It was shown that plants lacking a functional UDP-Glc transporter (AtUTr1) in the ER showed spontaneous activation of the UPR, and that responses triggering UPR induce the expression of this transporter [454]. Additionally, mutants of either *atutr1* or *atutr3* showed abnormalities in male and female germ line development. It was further shown that mutants of *atutr7*, also located in the ER, showed early proliferation of lateral roots as well as distorted root hairs when grown in the presence of high Suc [455]. It is thus tempting to speculate that under high Suc (high T6P), SnRK1 will be inactive, resulting in active protein synthesis and an effective secretory pathway. In such a case, *atutr1* mutants unable to import UDP-Glc might undergo normal protein synthesis under high Suc, but are unable to execute proper protein glycosylation, resulting in the observed growth defects in these pants. Additionally, a potential signaling role for UDP-Glc in plants cannot be excluded [456], since plants with abnormalities in the UDP-Glc metabolism show severe growth defects [457], and in some cases result in cell death [48,458].

Another interesting finding was that Suc can inhibit the GA-mediated degradation of DELLA (RGA; REPRESSOR OF GA1-3) in *Arabidopsis*, resulting in reduced seedling growth and anthocyanin accumulation [459]. Although T6P typically reflects Suc levels, controlling SnRK1 activity, it was shown that Suc-induced inhibition of DELLA degradation occurs independent of SnRK1 signaling pathways. DELLA proteins are master growth repressors. It has been shown that glycosylation of the DELLA protein RGA by the OGT SEC inhibits the binding of RGA to several of its interactors [320]. Consistent with this, sec-null mutants showed reduced GA responses and DELLA target gene expression, suggesting that O-GlcNAcylation of DELLA might fine-tune multiple downstream signaling events during plant development. It is thus tempting to speculate that sugar signaling, or at least Suc signaling might be responsible for the regulation of DELLA glycosylation/deglycosylation depending on the cellular sugar status. On the other hand, glycosylation of DELLA proteins might alter their sensitivity to degradation through Suc signaling. Such mechanisms would fine-tune the regulation of growth through DELLA according to sugar availability. Additionally, DELLA is mono-O-fucosylated by the novel OFT SPY in *Arabidopsis*, also resulting in reduced sensitivity to its upstream regulators [152]. The potential interplay between sugar signaling and glycosylation is further supported by the finding that SPY is responsible for fine-tuning the circadian clock in plants [154]. Whether and how O-fucosylation transduces nutrient signals or cellular energy homeostasis to the core clock machinery is still unclear. However, sugar signaling pathways might be an important intermediatory factor. Considering that AMPK and OGT in animals are highly interlinked [453], and can regulate one another, it is likely that such events are also conserved in plants.

Stress-mediated imbalances in plant source–sink relationships cause temporal mature leaf sweetening, contributing to the synthesis of antimicrobial compounds, some of them perhaps also able to counteract abiotic stresses (“Sweet Immunity” concepts) [42,307,460]. Evidence is accumulating that extracellular spraying of carbohydrates (e.g., priming with fructans) changes the intracellular sugar signaling context, leading to disease protection [43]. Additionally, ABA priming in fava bean altered the ratio of hexoses/Suc, and improved photosynthesis and growth [461].

Interestingly, a recent study showed that exogenous treatment with chitosan oligosaccharides resulted in major shifts in the accumulation of nucleotide sugars (precursors for glycosylation) and the expression of glycosylation related genes to confer pathogen resistance [462]. Enhanced levels of nucleotide sugars likely suggest a higher need for substrate for glycosylation reactions. Since it was shown that N-glycosylation mutants were still perceptive to priming, it is possible that O-glycosylation events are responsible for the primed effect as a recent report showed that O-glycosylation was important in the trade-off between growth and defense during systemic acquired resistance [463]. It is possible that intracellular sugar signaling events might be interconnected with altered glycosylation patterns observed in these plants. For instance, the accumulation of soluble sugars might reflect energy availability in primed plants during infection, allowing for normal protein synthesis and a functional secretory pathway, whereas in control treated plants, reduced soluble sugars might inhibit glycosylation to conserve energy levels [448]. In addition, enhanced soluble sugars can provide the building blocks required for the synthesis of nucleotide sugars, acting as the substrates for glycosylation reactions.

Thus, small soluble sugars might play a similar role during the different developmental stages of plants, controlling glycosylation according to sugar availability. On the other hand, alterations in glycosylation might have a severe effect on the sugar status of plants. A recent study showed that *Arabidopsis* plants disturbed in N-glycosylation pathways showed a significant reduction in photosynthetic capability [464], likely resulting in reduced soluble sugar accumulation. Such interplay between sugar signaling events (translating the cellular energy levels) and glycosylation might be important to balance the rate of glycosylation with the current sugar status. Another interesting study showed that expression of the *Coprinopsis cinerea* lectin 2 (CCL2), a fucoside-binding lectin from *Botrytis cinerea* in *Arabidopsis*, enhanced growth and disease resistance against a variety of pathogens [465]. When the carbohydrate binding ability of CCL2 was disturbed, disease tolerance is abolished, suggesting that carbohydrate interaction is required for induced tolerance. It was proposed that CCL2 enhances plant immunity by interacting with glycoproteins or glycosylated compounds involved in the regulation of plant immunity. This is supported by the finding that α-1,3-fucosylated glycans play an important role in plant immunity, affecting for instance the glycosylation of plant immune receptors [466]. It can be hypothesized that CCL2 interacts with the carbohydrate chain of plant immune receptors to activate immune responses.

## 7. Perspectives

Glycosylation of proteins and lipids represents an important and complex post-translational modification governing a broad variety of developmental processes, regulation of biotic and abiotic stress responses, as well as signaling pathways. Over the last decades, particular attention was paid to all aspects of N-glycosylation. However, recent findings related to O-glycosylation point out that there are still missing links that prevent a profound understanding. Whereas the N-glycosylation pathway as well as the nature and significance of N-glycoproteins are well-studied, more detailed studies are needed to reach the same level of comprehension for the O-glycosylation process and O-glycoproteins, but also to understand the interplay between both N- and O-glycosylation and the link with sugar and hormone signaling. There is more to investigate concerning the structure, distribution and evolutionary aspects of glycoproteins and glycolipids across plants, and interactomics at the level of specific pathways and processes during particular developmental stages.

Although sugars are important during glycosylation of plant proteins by functioning directly as the substrates for glycosylation, new evidence is emerging that interplay between sugar signaling events and glycosylation might also exist. At this stage, it is not clear if there is a direct correlation between cellular sugar levels and the level of glycosylation. However, with numerous findings showing differential glycosylation patterns during stress conditions, and the importance of sugar signaling events to alleviate stresses it is likely that a high level of communication between these pathways exist. Of particular interest for future studies will be to determine how glycosylation of SnRK1 and/or TOR target proteins are affected, and whether such events can alter the regulation by these central kinases. Additionally, future studies should also look at glycosylation patterns in plants lacking functional SnRK1 and TOR complexes under a variety of growth conditions to establish if sugar signaling events act as upstream regulators of glycosylation.

## Figures and Tables

**Table 1:** Overview of N- and O-glycosylation-related enzymes whose mutants display relevant phenotypes for plant development.

Enzyme	Activity	Role during Glycosylation	Mutant	Phenotype	Sources
ALG10	α-1,2-glucosyltransferase	N-glycan precursor synthesis	*alg10*	Dwarfism	[118]
ALG11	α-1,2-mannosyltransferase		*lew3*	Aberrant distribution of glycans, reduced cellulose synthesis, abnormal cell walls, reduced fertility	[119]
APTG1	mannosyltransferase		*aptg1*	Pollen tube is unable to penetrate the style; embryo lethality	[120]
CYT1	GTP:α-D-mannose-1-phosphate guanylyltransferase		*cyt1*	Disrupted embryonal development	[121]
DPMS1	dolichol phosphate mannose synthase 1		*dpms1*	Embryo lethality, wrinkled seed coats	[122]
EVN	dolichol kinase		*evn*	Pollen degeneration before pollen germination	[123]
GlcNA.UT	GlcNAc-phosphate UDP transferase		*glcna.ut1/2*	Aberrant gametogenesis, reduced fertilization, shorter siliques	[124]
DGL1	Subunit of OST complex	En bloc transfer	*dgl1*	Altered cell wall composition, embryo lethality, reduced growth of hypocotyls and roots, dense packed root hairs	[125]
HAP6			*hap6*	Shorter pollen tube	[126]
OST3/6			*ost3/6*	Enhanced salt sensitivity resulting in decreased root growth and root swelling	[127,128,129]
STT3a			*stt3a*		
TUN	UDP-glycosyltransferase		*tun*	Pollen defects, premature pollen rupture	[123]
GCSI	α-glucosidase I	N-glycan processing in the ER	*gcs1*	Embryo lethality, wrinkled seed coats	[130,131]
GCSII	α-glucosidase II		*gcs2*, *rsw3*	Embryo lethality, radially swollen roots, modified seed set	[132,133]
MOGS	mannosyloligosaccharide glucosidase		*mogs*	Reduced growth of primary and lateral roots, abnormal root hair initiation and elongation	[134]
UGGT	UDP-glucose:glycoprotein glucosyltransferase	ERAD	*-*	No mutant	[135]
PNGase	peptide-N^4^-(N-acetyl-β-D-glucosaminyl) asparagine amidase		*-*	No mutant	[78,136,137]
ENGase	endo-N-acetyl-β-D-glucosaminidase	De-N-glycosylation	*-*	No mutant	[78,136,137]
α-MAN	α-mannosidase		*-*	No mutant	[138,139]
β-NAHase	β-D-N-acetylhexosaminidase		*-*	No mutant	[138,139]
FucT	α-1,3-fucosyltransferase	N-glycan maturation in the Golgi apparatus	*fuct*	Reduced seed set	[140]
FUCTc	α-1,4-fucosyltransferase		*fuctc*	Increased root hair length, early root hair elongation	[141]
GALT	β-1,3-galactosyl-transferase		*galt*	Increased root hair length, early root hair elongation	[141]
GnT1	N-acetylglucosaminyl transferase I		*gnt1*	Cell wall shortcomings: reduced thickness and cellulose content; lethality	[140,142]
				Necrosis on fruits, incomplete fruit ripening, early fruit drop	[143]
				Increased root hair length, early root hair elongation, salt sensitivity resulting in altered root tip morphology	[141,144]
MANII	α-mannosidase II		*manII*	Increased root hair length, early root hair elongation, salt sensitivity resulting in altered root tip morphology	[141]
MNS1	mannosidase I		*mns1*	Reduced germination capacity	[140]
MNS2			*mns1*, *mns2*, *mns3*	Shorter roots	[145]
MNS3					
XylT	β-1,2-xylosyltransferase		*rcn11*	Postponed seed germination	[140,146,147]
GFAT1	Glutamine:fructose-6-phosphate amidotransferase 1	Sugar delivery	*gfat1*	Aberrant pollen cell wall	[148]
USP	UDP-sugar pyrophosphorylase		*usp*	Infertile pollen, improper pollen sac development	[112]
ROCK1	UDP-GlcNAc transferase, UDP-GalNAc transferase		*rock1*	Cytokinin shortage-like phenotype, increased organ formation rate, increased UPR	[117]
GlcNAc.UT	UDP-GlcNAc transferase		*glcna.ut1*, *glcna.ut2*	Severe developmental shortcomings. Disturbed gametogenesis of male and female gametes. Embryonal problems, shorter siliques with fewer and shrunken seeds in double mutant	[149]
OFT	O-fucosyltransferase	O-glycan modification	*oft*	Impaired pollen tube penetration ability	[150]
SEC	O-GlcNAc transferase		*sec*	Nonviable seeds, decreased number of leaves	[90,151]
SPY	O-fucosyltransferase		*spy*	Germination in presence of germination inhibitors	[152]
				Enhanced trichome branching, reduced leaf serration size and number of leaf serrations	[153,154]
PSL	β-1,6-GlcNAc-transferase		*psl*	Early leaf senescence, enhanced ethylene production	[155]
BZ1	UDP-Gal/Glu epimerase		*bz1*	Abnormal cell walls, abnormal chloroplast membranes	[156]
P4H1	Prolyl-4-hydroxylase	Hydroxyproline production	*p4h1*	Lack of trichomes, large leaves	[157,158]
P4H2			*p4h2*	Short root hairs	[94,159,160]
P4H5			*p4h5*		
P4H13			*p4h13*		
P4H7			*p4h7*	Large leaves	[158]
P4H9			*p4h9*		
HPAT1	Hydroxyproline O-β-arabinosyltransferase	O-arabinosylation of extensins	*hpat1*	Decreased number of leaves, accelerated leaf senescence, short root hairs	[161,162]
HPAT2			*hpat2*		
HPAT3			*hpat3*		
RRA1	β-1,2-arabinosyltransferase		*rra1*	Short root hairs	[95]
RRA2			*rra2*		
RRA3			*rra3*		
XEG113	β-1,2-arabinosyltransferase		*xeg113*	Larger petioles, short root hairs	[95,163]
ExAD	α-1,3-arabinosyltransferase			Short root hairs	[164]
SGT1	Serine O-α-galactosyltransferase	O-galactosylation of extensins	*sgt1*	Larger leaf rosette, longer roots	[91]
RAY1	β-arabinofuranosyltransferase	O-arabinosylation of arabinogalactan proteins	*ray1*	Short primary root, reduced leaf rosette size	[165]
GALT2	Hydroxyproline-O-β-galactosyltransferase	O-galactosylation of arabinogalactan proteins	*galt2*	Reduced seed coat mucilage, reduced seed set; pleiotropic effects on roots, leaves and root hairs in different single or multiple mutants	[162,166,167,168,169]
GALT3			*galt3*		
GALT4			*galt4*		
GALT5			*galt5*		
GALT6			*galt6*		
HPGT1			*hpgt1*		
HPGT2			*hpgt2*		
HPGT3			*hpgt3*		
GLCAT14a	β-1,6-glucuronosyltransferase	O-glucuronidation of arabinogalactan proteins	*glcat14a*	Reduced trichome size, branching in double (*glcat14a*/*glcat14b*) and triple mutant	[169]
GLCAT14b			*glcat14b*		
GLCAT14c			*glcat14c*		

## Data Availability

No new data were created or analyzed in this study. Data sharing is not applicable to this article.
